# CNS-targeted base editing of the major late-onset Tay-Sachs mutation alleviates disease in mice

**DOI:** 10.1172/JCI183434

**Published:** 2025-06-17

**Authors:** Maria L. Allende, Mari Kono, Y. Terry Lee, Samantha M. Olmsted, Vienna Huso, Jenna Y. Bakir, Florencia Pratto, Cuiling Li, Colleen Byrnes, Galina Tuymetova, Hongling Zhu, Cynthia J. Tifft, Richard L. Proia

**Affiliations:** 1 Genetics and Biochemistry Branch, National Institute of Diabetes and Digestive and Kidney Diseases, and; 2 Medical Genetics Branch, National Human Genome Research Institute, NIH, Bethesda, Maryland, USA.

**Keywords:** Genetics, Neuroscience, Genetic diseases, Monogenic diseases

## Abstract

Late-onset Tay-Sachs (LOTS) disease is a lysosomal storage disorder most commonly caused by a point mutation (c.805G>A) in the *HEXA* gene encoding the α subunit of the lysosomal enzyme β-hexosaminidase A. LOTS manifests as a range of gradually worsening neurological symptoms beginning in young adulthood. Here, we explored the efficacy of an adenine base editor (ABE) programmed with an sgRNA to correct the *HEXA* c.805G>A mutation. Base editing in fibroblasts from a patient with LOTS successfully converted the pathogenic *HEXA* c.805A to G and partially restored β-hexosaminidase activity, with minimal genome-wide off-target editing. We generated a LOTS mouse model in which the mice exhibited decreased β-hexosaminidase activity, accumulation of GM2 ganglioside in the brain, progressive neurological manifestations, and reduced lifespan. Treatment of LOTS mice with the neurotropic virus AAV-PHP.eB carrying the ABE and an sgRNA targeting the LOTS point mutation partially corrected the c.805G>A mutation in the CNS, significantly increased brain β-hexosaminidase activity, and substantially reduced GM2 ganglioside accumulation in the brain. Moreover, the therapy delayed symptom onset and significantly extended median lifespan. These findings highlight the potential of base editing as an effective treatment for LOTS and its broader applicability to other lysosomal storage disorders.

## Abstract 2

Base editing corrected a Tay-Sachs mutation in mice, restored HEXA activity, reduced GM2 ganglioside accumulation, improved neurological function, and extended lifespan.

## Graphical abstract

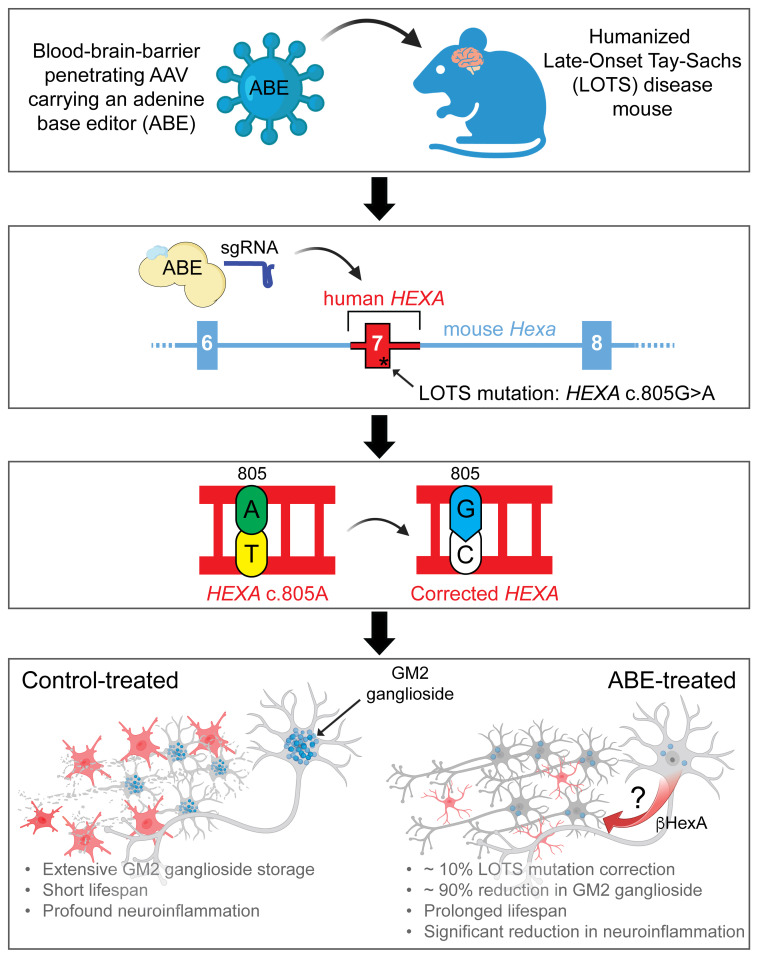

## Introduction

Tay-Sachs disease is a rare autosomal recessive lysosomal storage disorder resulting from mutations in the *HEXA* gene (1, 2). This gene encodes the α subunit of the lysosomal enzyme β-hexosaminidase A. The clinical severity of Tay-Sachs disease occurs along a spectrum that is based on the amount of residual β-hexosaminidase A enzyme activity present. The deficiency of β-hexosaminidase A results in the lysosomal accumulation of its substrate, GM2 ganglioside, an intermediate in the degradation of higher order gangliosides that is abundant in neurons. Null mutations in *HEXA* resulting in a complete loss of β-hexosaminidase A activity lead to an acute infantile-onset neurodegenerative disease. *HEXA* mutations allowing residual β-hexosaminidase A activity result in less severe forms of the disease, known as juvenile-onset and late-onset Tay-Sachs (LOTS) disease.

Like the other forms of Tay-Sachs disease, LOTS pathophysiology stems from the accumulation of GM2 ganglioside, which damages neurons and results in disease manifestations (1, 3, 4). The symptoms of LOTS are variable and can include cerebellar ataxia, muscle weakness, peripheral neuropathy, and psychiatric symptoms. However, unlike the more severe infantile-onset and juvenile-onset forms of Tay-Sachs disease, LOTS typically does not affect life expectancy and progresses much more slowly (1, 3, 5). Most patients with LOTS carry an α subunit glycine to serine amino acid change at position 269, resulting from a G to A transition (c.805G>A) in exon 7 of the *HEXA* gene (6, 7). In patients with LOTS, *HEXA* alleles carrying the c.805G>A point mutation are found either in combination with other deleterious *HEXA* alleles or in a homozygous state (8, 9).

Base editing is a form of genome editing that shows promise as a durable genetic therapy for the correction of pathogenic point mutations (10). Adenine base editors (ABEs), consisting of a modified adenine deaminase linked to a Cas9 nickase, are directed to specific genomic locations by an sgRNA. The ABE modifies G•C to A•T base pairs within a small editing window without, importantly, causing dsDNA breaks.

In this study, we successfully corrected the common LOTS *HEXA* c.805G>A mutation in both fibroblasts from a patient with LOTS and in a LOTS mouse model using ABE treatment. In LOTS fibroblasts, approximately 80% mutation correction was achieved, with minimal genome-wide off-target editing. Further, α subunit–specific β-hexosaminidase activity was restored to approximately 50% of the level seen in control human fibroblasts. Moreover, a single intravenous injection of a neurotropic AAV vector, carrying the ABE and LOTS-sgRNA, led to partial correction of the c.805G>A mutation in the CNS of adult LOTS mice. This genetic correction increased brain β-hexosaminidase activity, reduced GM2 ganglioside accumulation in the brain, and decreased brain expression of neuroinflammation markers. Most notably, the base-editor treatment delayed the onset of disease symptoms and prolonged the lifespan of these mice compared with what was observed in control-treated mice.

These results support the potential of base editing as a therapeutic approach to permanently correct the LOTS mutation in the CNS. Furthermore, they suggest broader applicability of base editing for treating a range of lysosomal storage disorders.

## Results

### Base editing corrects the HEXA c.805G>A mutation in fibroblasts from a patient with LOTS.

**Figure 1:**
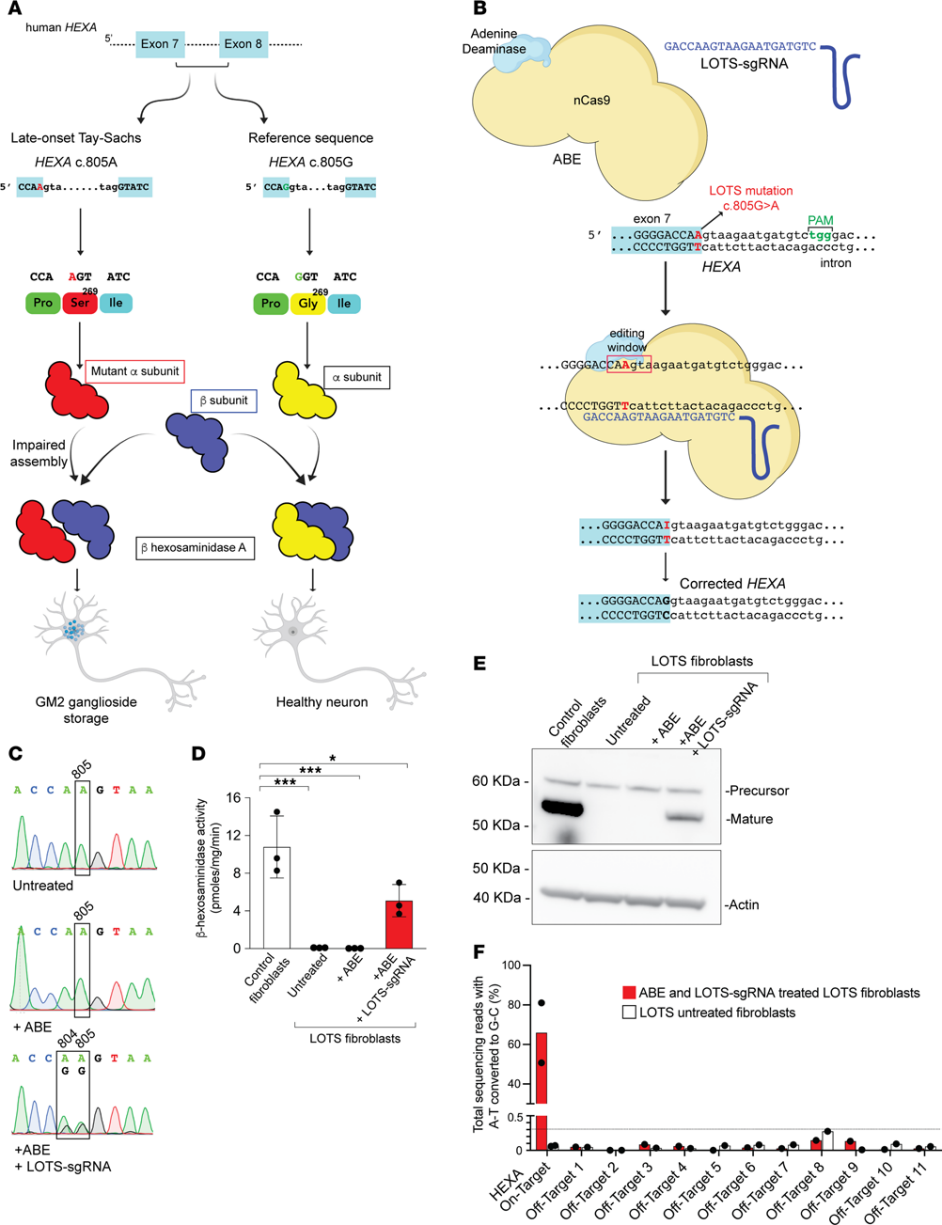
Base editing corrects the *HEXA* c.805G>A mutation in fibroblasts from a patient with LOTS. (**A**) Schematic of late-onset Tay-Sachs (LOTS) disease. A point mutation in the last base of exon 7 of *HEXA* (c.805G>A) causes a Gly269Ser substitution in the α subunit, impairing assembly of β-hexosaminidase A, the lysosomal enzyme that degrades GM2 ganglioside. Reduced enzymatic activity leads to GM2 ganglioside accumulation in neuronal lysosomes. (**B**) Base-editing strategy. An adenine base editor (ABE), composed of Cas9 nickase fused to an adenine deaminase, was programmed with a LOTS-sgRNA targeting the *HEXA* c.805G>A mutation. Editing converted A-to-G within the specified editing window (red box), correcting the LOTS *HEXA* c.805G>A mutation. (**C**–**E**) Base editing in fibroblasts from a patient with LOTS. Fibroblasts homozygous for *HEXA* c.805G>A were transduced with lentivirus encoding ABE only (+ABE), both ABE and LOTS-sgRNA (+ABE, +LOTS-sgRNA), or left untreated and cultured for 4 weeks. Sanger sequencing of PCR amplicons confirmed targeted base editing (**C**). (**D**) β-Hexosaminidase A activity was assayed in cell extracts (mean ± SD, *n* = 3). Control fibroblasts from an unaffected individual were used as a positive control. Statistical significance was determined by 1-way ANOVA with Bonferroni’s correction (*P < 0.05, ***P < 0.001). (**E**) Western blot of α subunit expression in edited fibroblasts. Precursor and mature forms are indicated; β-actin was used as loading control. (**F**) Off-target analysis. CIRCLE-Seq identified 11 candidate off-target loci, which were amplified and deep-sequenced in fibroblasts transduced with ABE and LOTS-sgRNA (cultured 27 weeks), and in untreated controls. Shown are percentages of A-to-G conversions at each locus, including the on-target site (*HEXA* c.805A) for comparison. Partially created with BioRender.com.

Most patients with LOTS carry a point mutation (c.805G>A) in the *HEXA* gene situated at the 3′ end of exon 7 (Figure 1A) (3, 8). This mutation results in an amino acid substitution, changing glycine to serine at position 269 of the α subunit polypeptide. Consequently, this alteration limits the formation of the active enzyme β-hexosaminidase A, which is a heterodimer consisting of an α subunit and a β subunit, the latter of which is encoded by the *HEXB* gene (11, 12). The mutation causes enzyme levels to be reduced below the threshold necessary for the effective breakdown of GM2 ganglioside in the lysosomes of neurons, resulting in GM2 ganglioside accumulation.

To determine the feasibility of base editing to correct the common LOTS *HEXA* c.805G>A point mutation, we derived skin fibroblasts from a patient with LOTS who was homozygous for this point mutation. Using a canonical protospacer adjacent motif (PAM) site, the pathogenic *HEXA* c.805G>A mutation was positioned within the editing window of the ABE at protospacer position 6 using an sgRNA (LOTS-sgRNA) (Figure 1B). Noteworthy is the presence of an adjacent A at position c.804 (protospacer position 5).

The fibroblasts from the patient with LOTS were transduced with a lentivirus carrying the ABE alone or with a lentivirus carrying the ABE along with a second lentivirus to express the LOTS-sgRNA. After 4 weeks of culture, the *HEXA* genomic region containing the c.805G>A mutation was PCR amplified and then Sanger sequenced (13). The *HEXA* gene sequence from the fibroblasts from the patient, either untreated or transduced with ABE lentivirus without the LOTS-sgRNA, contained only the mutant A at position c.805. In contrast, the *HEXA* gene sequence from LOTS fibroblasts transduced with both the ABE and LOTS-sgRNA lentiviruses showed both A and G at position c.805, demonstrating a partial correction of the LOTS mutation (Figure 1C). The adjacent A at position c.804 also showed conversion to G; however, this resulted in a synonymous substitution, converting the proline codon at position 268 of *HEXA* from CCA to CCG. These results showed that ABE and LOTS-sgRNA treatment resulted in a partial correction of the pathogenic *HEXA* c.805 mutation in cultured fibroblasts from a patient with LOTS without introducing consequential changes near the target site.

To determine the degree of restoration of functional *HEXA* after base editing, we measured α subunit–specific β-hexosaminidase enzyme levels utilizing a fluorometric substrate that is specific for the catalytic site of the α subunit (Figure 1D). Both untreated LOTS fibroblasts and those transduced with lentivirus encoding only the ABE without the LOTS-sgRNA exhibited negligible α subunit–specific β-hexosaminidase A activity. In contrast, LOTS fibroblasts transduced with both the ABE and LOTS-sgRNA lentiviruses displayed α subunit–specific β-hexosaminidase A activity at approximately 50% of the level found in control human fibroblasts, consistent with the observed correction of the pathogenic LOTS mutation (Figure 1, C and D). The mutation correction resulted in the presence of the mature lysosomal form of the β-hexosaminidase α subunit in the fibroblasts from the patient with LOTS, consistent with the observed increase in enzymatic activity (Figure 1E) (12). These findings indicate that base editing can partially correct the *HEXA* c.805G>A mutation and functionally restore α subunit–specific β-hexosaminidase activity in cells derived from a patient with LOTS.

To identify the potential genome-wide off-target sites of the ABE, we utilized CIRCLE-Seq to first map the genome-wide off-target cleavage sites of Cas9 directed by the LOTS-sgRNA on human genomic DNA (14, 15) (Supplemental Figure 1 and Supplemental Table 1; supplemental material available online with this article; https://doi.org/10.1172/JCI183434DS1). After identifying candidate Cas9-recognition sites, we selected 11 loci and generated PCR amplicons of these loci from genomic DNA isolated from LOTS fibroblasts cultured for 7 months after transduction with lentiviruses carrying the ABE and LOTS-sgRNA, as well as untreated LOTS fibroblasts. Upon deep sequencing those amplicons, we found that despite achieving approximately 80% on-target editing efficiency in the long-term cultured ABE plus LOTS-sgRNA–transduced fibroblasts, only negligible A>G conversion — not exceeding 0.15% — was observed at any of the candidate loci in ABE plus LOTS-sgRNA–transduced LOTS fibroblasts. The level of these conversion events did not exceed those of the control fibroblasts (Figure 1F).

### Generation of a LOTS mouse model for base editing.

**Figure 2:**
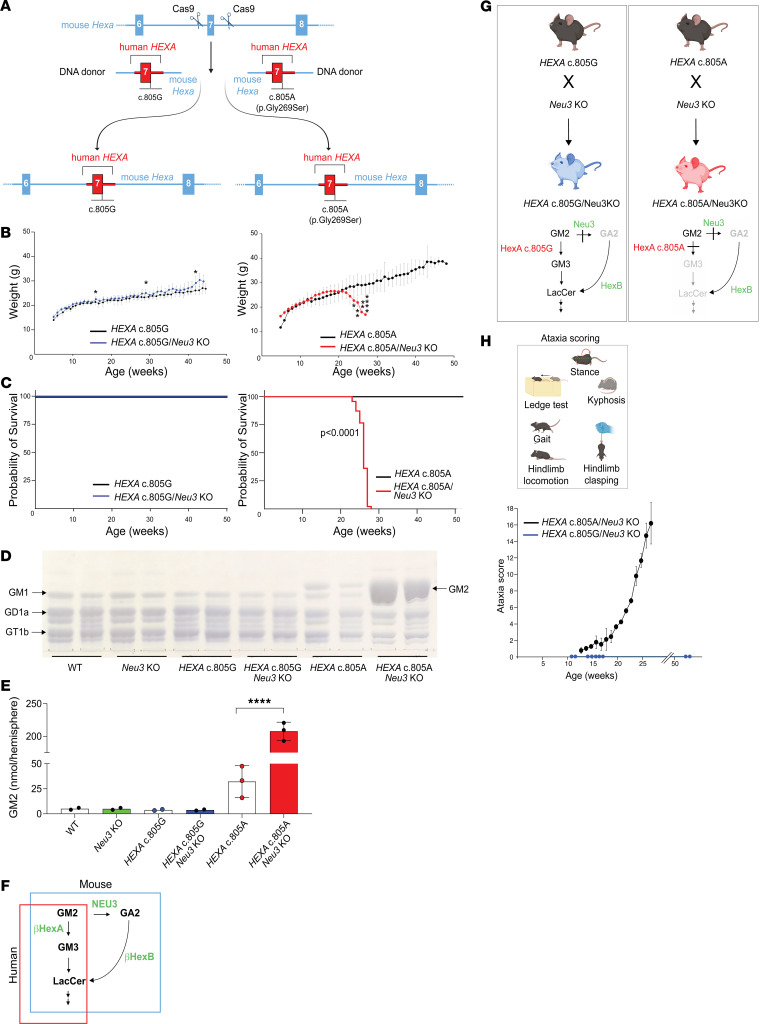
Generation of a LOTS mouse model for base editing. (**A**) Schematic of the engineered *Hexa* locus. Cas9-mediated targeting inserted human *HEXA* exon 7 and flanking intron sequences into the mouse genome to generate either the reference allele (*HEXA* c.805G, left) or LOTS allele (*HEXA*c.805A, right). Mouse sequences are in blue; human sequences in red. (**B**) Body-weight progression in female mice. Weekly weights were recorded for *HEXA* c.805G and *HEXA* c.805G/*Neu3-*KO (left) and *HEXA* c.805A and *HEXA* c.805A/*Neu3-*KO mice (right). Data are mean ± SD (*n* = 10–15). *P < 0.05, **P < 0.01 (Student’s *t* test). (**C**) Kaplan-Meier survival curves for *HEXA* c.805G and *HEXA* c.805G/*Neu3-*KO (left) and *HEXA* c.805A and *HEXA*c.805A/*Neu3-*KO mice (right). Combined sexes shown (*n* = 25–57). (**D** and **E**) GM2 ganglioside levels in brain. Gangliosides were extracted from female mice aged 24–26 weeks (*n* = 2–3/genotype) and analyzed by high-performance TLC. (**D**) Representative high-performance TLC plate; each lane contains 5% of gangliosides from 1 brain hemisphere. Arrows indicate ganglioside standards. (**E**) Quantification of GM2 band intensities (mean ± SD). Each dot represents data from 1 mouse. ****P < 0.0001 (Student’s *t* test). (**F**) GM2 degradation pathways. In humans (red box) and mice (blue box), β-hexosaminidase A converts GM2 to GM3. In mice only, NEU3 also degrades GM2 to GA2, bypassing β-hexosaminidase A. (**G**) Generation of control and LOTS mice. *HEXA* c.805G or c.805A mice were crossed with *Neu3-*KO mice to generate *HEXA* c.805G/*Neu3-*KO (control) and *HEXA* c.805A/*Neu3-*KO (LOTS) mice. Diagrams show expected GM2 degradation in each line. (**H**) Neurological evaluation. Mice were scored weekly on 6 criteria (left box) starting at 7 weeks. Mean ± SD shown (*n* = 16 LOTS, *n* = 27 controls; sexes combined). Partially created with BioRender.com.

We engineered a humanized LOTS mouse model that contained the *HEXA* c.805G>A mutation in the context of human genomic DNA sequences to enable the testing of base editors that target authentic human genomic *HEXA* sequences. Through CRISPR/Cas9 genomic editing, we excised a 240-bp segment of the mouse *Hexa* gene encompassing exon 7 and flanking intron sequences and replaced it with the corresponding segment of the human *HEXA* gene. Two mouse lines were established with human exon 7 and flanking intron sequences, one with the reference G at *HEXA* c.805 (*HEXA* c.805G) and the other with the mutant A at *HEXA* c.805 (*HEXA* c.805A) (Figure 2A).

Mice homozygous for the *HEXA* c.805 G>A point mutation (*HEXA* c.805A mice) exhibited significantly lower levels of α subunit β-hexosaminidase enzyme activity in the brain compared with both WT C57BL/6 mice and mice homozygous for human *HEXA* exon 7 carrying c.805G (*HEXA* c.805G mice) (Supplemental Figure 2A), consistent with the pathogenic nature of the mutation. Both the *HEXA* c.805G/*Neu3-*KO and the *HEXA* c.805A/*Neu3-*KO mice expressed β-hexosaminidase A precursor and mature forms of the α subunit polypeptide (Supplemental Figure 2B). However, both homozygous mouse lines (*HEXA* c.805G and *HEXA* c.805A) survived past 1 year and maintained similar body weights over the study period (Figure 2, B and C, and Supplemental Figure 2C). The *HEXA* c.805A mice but not the *HEXA* c.805G mice demonstrated a slight elevation of GM2 ganglioside levels in the brain compared with WT mice (Figure 2, D and E).

The absence of a severe phenotype in mice carrying the LOTS *HEXA* c.805A mutation was not unexpected. Unlike humans, mice with disrupted *Hexa* alleles are only mildly affected due to an alternative GM2 ganglioside degradation pathway (Figure 2F) (16). Both humans and mice possess a β-hexosaminidase A–mediated pathway for GM2 ganglioside degradation. However, a second degradation pathway exists uniquely in mice. This mouse-specific pathway involves sialidase NEU3, which processes GM2 ganglioside into GA2 glycolipid (Asialo-GM2-ganglioside) (17). β-Hexosaminidase B, the product of the intact *Hexb* gene, can now act on GA2 glycolipid to form lactosylceramide, bypassing the block caused by the absence of β-hexosaminidase A. To create a symptomatic LOTS model, we established mice homozygous for the *HEXA* c.805A mutation on a *Neu3-*KO background, thereby disabling the mouse-specific bypass pathway (Figure 2G). In these mice then, like in humans, GM2 ganglioside catabolism would rely primarily on β-hexosaminidase A.

The *HEXA* c.805G/*Neu3-*KO mice with the normal human *HEXA* sequence displayed WT levels of α subunit–specific β-hexosaminidase activity. However, the *HEXA* c.805A/*Neu3-*KO mice carrying the LOTS point mutation showed significantly lower α subunit–specific β-hexosaminidase activity (Supplemental Figure 2A). Both the *HEXA* c.805G/*Neu3-*KO and the *HEXA* c.805A/*Neu3-*KO mice expressed β-hexosaminidase A precursor and mature forms of the α subunit polypeptide (Supplemental Figure 2B). These mice underwent weekly evaluations, including body-weight measurements and evaluation of ataxia. *HEXA* c.805A/*Neu3-*KO mice began to lose weight at about 22 weeks of age (Figure 2B and Supplemental Figure 2C) and had a lifespan of approximately 26–27 weeks (Figure 2C). Their brains accumulated GM2 ganglioside compared with WT mouse brains (Figure 2, D and E), and ataxia symptoms emerged at around 13 weeks of age (Figure 2H). In contrast, *HEXA* c.805G/*Neu3-*KO animals, which harbored the normal human *HEXA* exon 7 and flanking intron sequences, did not display early weight loss (Figure 2B and Supplemental Figure 2C) or ataxic symptoms (Figure 2H) and lived beyond 50 weeks (Figure 2C). In addition, these *HEXA* c.805G/*Neu3-*KO mice did not show abnormally high GM2 ganglioside levels in the brain (Figure 2, D and E). In summary, the results establish the *HEXA* c.805A/*Neu3-*KO mice as a model of LOTS that incorporates the *HEXA* c.805G>A pathogenic variant together with flanking human DNA sequences. This model is characterized by a deficiency in α subunit–specific β-hexosaminidase activity, GM2 ganglioside storage in the brain, ataxia, weight loss, and a substantially reduced lifespan. Although the mouse phenotype is more severe than that expected for LOTS disease, its distinct features provide measurable endpoints for evaluating base editing as a potential therapeutic approach. Mice homozygous for the LOTS pathogenic mutation on the *Neu3-*KO background (*HEXA* c.805A/*Neu3-*KO) are hereafter referred to as “LOTS mice.” The lack of disease symptoms in the *HEXA* c.805G/*Neu3-*KO mice, which carried the WT *HEXA* gene sequences, demonstrated that the hybrid *HEXA*/*Hexa* gene was functional and that correction of the *HEXA* c.805G>A mutation in LOTS mice should restore *HEXA* function and potentially alleviate the disease features associated with the mutation.

### Base-editor treatment corrects the HEXA c.805G>A mutation in the brain and spinal cord and partially restores β-hexosaminidase activity in LOTS mice.

Our strategy for the therapeutic rescue of the LOTS mice by base editing involved delivering the ABE and LOTS-sgRNA to the CNS using adeno-associated virus (AAV). AAV vectors are currently being utilized in gene-therapy trials for treating lysosomal storage diseases, including Tay-Sachs disease (18). Specifically, we employed the PHP.eB serotype of AAV, known to effectively cross the blood-brain barrier to infect brain cells, including neurons (19, 20).

To accommodate the large size of the ABE sequence, we implemented a dual AAV system. The N-terminal portion of the ABE sequence was carried in one AAV, while the C-terminal portion of ABE and the LOTS-sgRNA sequences were contained in the other AAV. Split inteins, attached to the 2 ABE segments, catalyzed protein trans-splicing (21), facilitating the formation of the full-length ABE (Supplemental Figure 3A).

**Figure 3:**
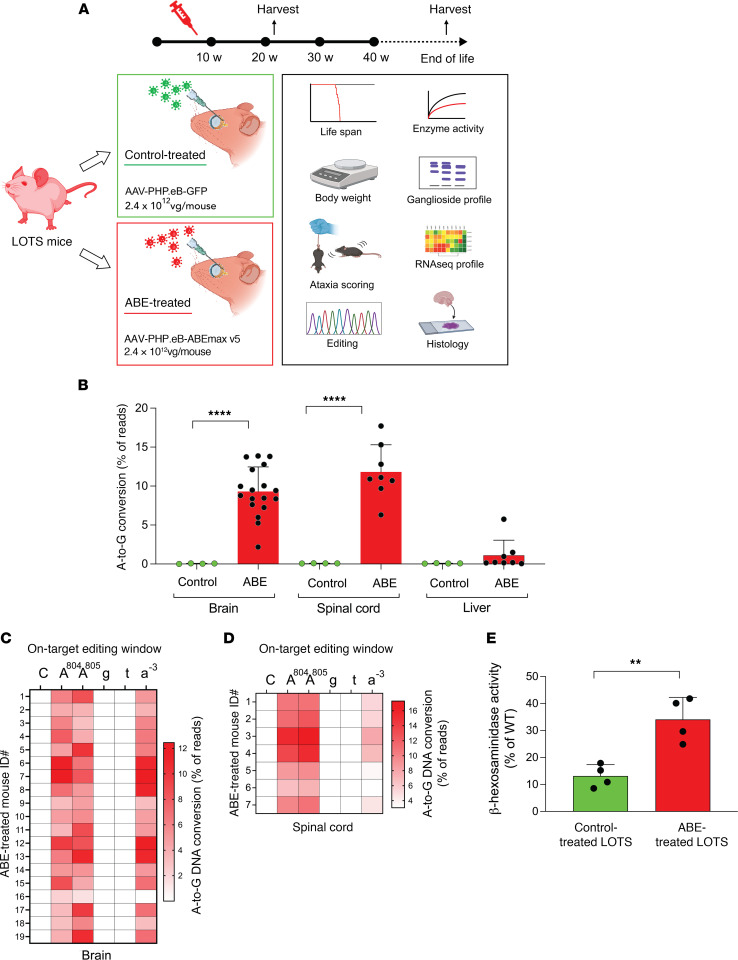
Base-editor treatment corrects the *HEXA* c.805G>A mutation and partially restores brain β-hexosaminidase activity in LOTS mice. (**A**) Schematic of ABE treatment in LOTS mice. At 6–7 weeks of age, LOTS mice were injected retro-orbitally with either AAV-PHP.eB-GFP (control) or a 1:1 mixture of AAV-PHP.eB vectors carrying v5 AAV-ABE N- and C-terminal components with LOTS-sgRNA (ABE-treated). Total dose was 2.4 × 10^12^ vg/mouse. Tissues were collected at 21 weeks or end of life. (**B**) Base editing at the *HEXA* c.805A site. Editing efficiency was quantified in brain, spinal cord, and liver DNA from control- and ABE-treated LOTS mice at 21 weeks using next-generation sequencing. A-to-G conversion was expressed as mean ± SD (brain: *n* = 18 ABE, *n* = 4 control; spinal cord/liver: *n* = 8 ABE, *n* = 4 control). Each dot represents 1 mouse. ****P < 0.0001 by 1-way ANOVA with Bonferroni’s correction. (**C** and **D**) Heatmaps of A-to-G conversion across the ABE editing window in brain (**C**) and spinal cord (**D**) from ABE-treated mice. Each row represents 1 mouse. Protospacer sequence is shown above; uppercase: exon, lowercase: intron. (**E**) β-Hexosaminidase activity in brain lysates from 21-week-old WT, control-treated LOTS, and ABE-treated LOTS mice. α subunit–specific activity is expressed as a percentage of WT (set at 100%). Mean ± SD shown (*n* = 4 per group, males). **P < 0.01 by Student’s *t* test. Partially created with BioRender.com.

Male and female LOTS mice at 6–7 weeks of age were retro-orbitally injected, to achieve intravenous delivery, with dual AAV-PHP.eB virus (2.4 × 10^12^ vg) carrying the split ABE and LOTS-sgRNA (Figure 3A). These mice were termed ABE treated. For controls, male and female LOTS mice were injected with AAV-PHP.eB encoding GFP (2.4 × 10^12^ vg). These mice were termed control treated. Mice were monitored weekly for ataxia symptoms, and a group of ABE-treated and control-treated LOTS mice were euthanized at 21 weeks and evaluated for base-editor expression, base-editing efficiency, α subunit–specific β-hexosaminidase activity, brain GM2 ganglioside accumulation, brain gene expression, and brain immunohistology (Figure 3A and Supplemental Table 2). A second group of ABE-treated and control-treated LOTS mice were monitored for lifespan, body weight, and ataxia until end of life.

Western blot analysis of brain extracts from ABE-treated mice, using an antibody directed against the N-terminal portion of Cas9, revealed a band corresponding in size to full-length ABE, indicating successful intein-mediated protein splicing (Supplemental Figure 3B).

To evaluate the extent of full-length ABE expression in the brain, we employed the proximity ligation assay (PLA), a technique that detects posttranslational protein modifications (22). Specifically, we utilized 2 distinct Cas9 antibodies — one targeting the C-terminal of Cas9 (encoded by one AAV) and the other targeting the N-terminal of Cas9 (encoded by the second AAV). The assay involves DNA primers covalently linked to these antibodies, followed by a hybridization step and DNA amplification with fluorescent probes. Fluorescent signals are then visualized as proximity spots under a microscope. A positive signal occurs when the 2 antibodies are within less than 40 nm of each other, confirming successful reconstitution of full-length ABE.

We applied PLA to brain sections from both control-treated and ABE-treated mice, quantifying the number of DAPI-positive cells that also exhibited PLA positivity (Supplemental Figure 3C). PLA signals were distributed throughout the brain: approximately 50% of cells in the cortex and thalamus showed editor positivity; around 20% in the midbrain, pons, and medulla; and approximately 10% in the cerebellum (Supplemental Figure 3D). The widespread expression of reconstituted ABE was consistent with the brain-wide distribution of GFP expression in mice injected with AAV-PHP.eB encoding GFP (Supplemental Figure 3E).

To quantify the extent of correction of the *HEXA* c.805G>A mutation in the ABE-treated LOTS mice, genomic DNA was obtained from the brain, spinal cord, and liver, and the genomic region carrying the mutation was PCR amplified and subjected to next-generation sequencing. In the brain of ABE-treated mice, the correction of the c.805G>A mutation occurred at a mean frequency of 9.3%. Spinal-cord tissue in ABE-treated mice exhibited a similar level of mutation correction (11.8%). Meanwhile, liver DNA in ABE-treated mice showed a mean correction frequency of the c.805G>A mutation of only 1.1%, reflecting the preferential targeting of the CNS by the PHP.eB serotype of AAV (Figure 3B). Bystander editing was observed at c.804A, resulting in a synonymous change (proline codon CCA to CCG). Editing was also noted in the intron (IVS7) at c.805-3A (Figure 3, C and D). The control-treated LOTS mice did not show evidence of base conversion in any of the tissues examined (Figure 3B).

We analyzed 7 brain regions in ABE-treated and control-treated LOTS mice at 21 weeks of age: the cortex; hippocampus; thalamus and hypothalamus; cerebellum; medulla, pons, midbrain, and superior colliculus; spinal cord; and sciatic nerve. Our findings showed that all analyzed brain regions in ABE-treated LOTS mice exhibited similar levels of mutation editing, except for the cerebellum, which displayed significantly lower editing efficiency compared with other regions (Supplemental Figure 4A). Editing in the sciatic nerve was minimal, consistent with previous reports indicating the low tropism of AAV-PHP.eB for the peripheral nervous system (20). In addition, microglia, a cell type central to the pathogenesis of the GM2 gangliosidoses, showed no evidence of editing (Supplemental Figure 4B), consistent with the generally refractory nature of this cell type to AAV-mediated expression of target genes (23).

To assess the functional restoration of the *HEXA* gene, we evaluated α subunit–specific β-hexosaminidase activity in brain extracts from 21-week-old ABE-treated and control-treated LOTS mice. Control-treated LOTS mouse brain exhibited approximately 10% of WT enzyme activity (Figure 3E). In contrast, ABE-treated LOTS mice demonstrated significantly higher levels of α subunit–specific β-hexosaminidase activity in the brain, with a mean activity level of 34% of WT levels (Figure 3E).

### Base-editor treatment reduces GM2 ganglioside accumulation in the brain of LOTS mice.

**Figure 4:**
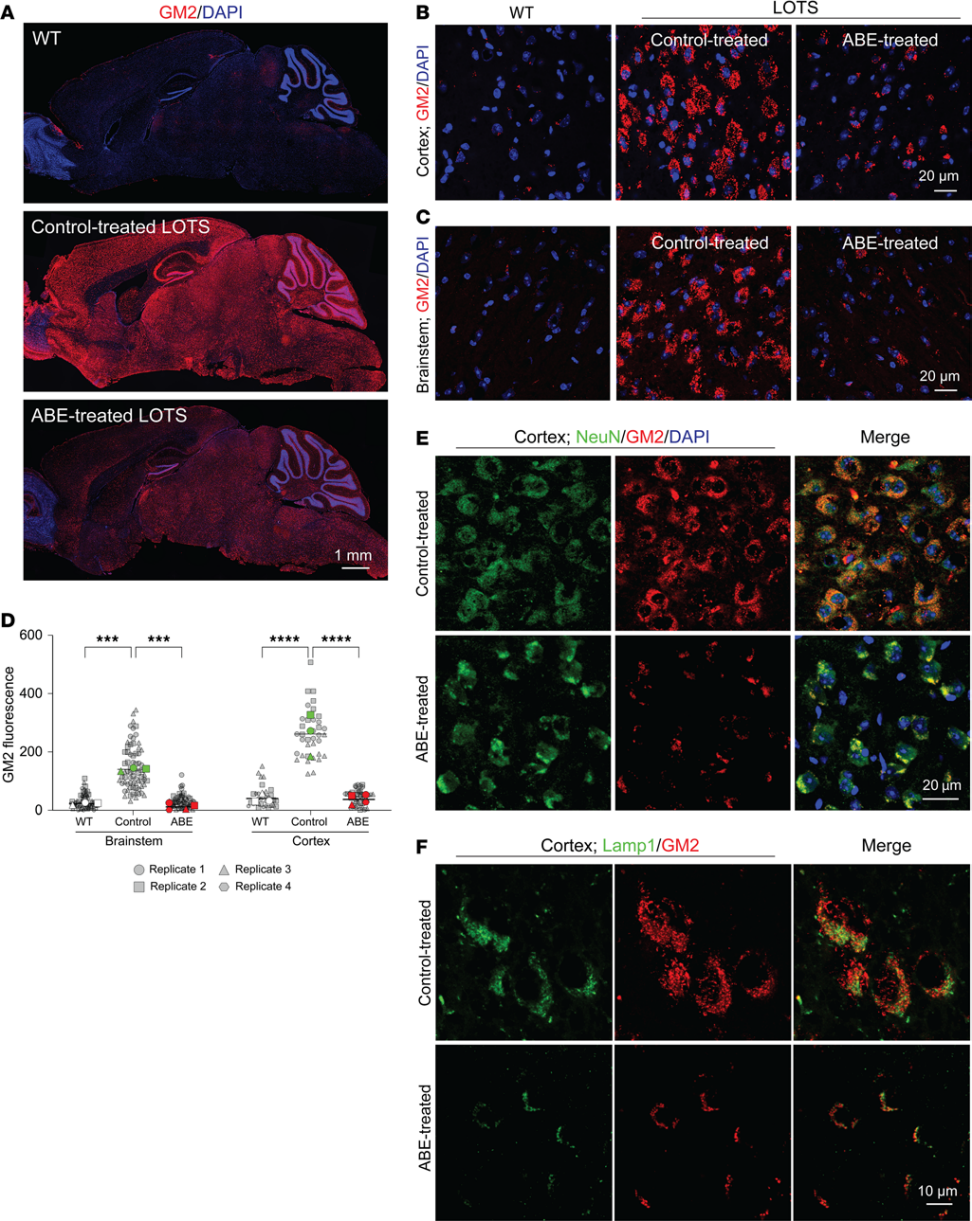
Base-editor treatment reduces brain GM2 ganglioside accumulation in LOTS mice. WT, control-treated LOTS, and ABE-treated LOTS mice were euthanized at 21 weeks of age, and sagittal brain sections were prepared (*n* = 4 per group; mixed males and females). The AAV-treated mice each received 2.4 × 10^12^ vg. (**A**) Representative sagittal brain sections stained with anti-GM2 ganglioside antibody (red) and counterstained with DAPI (blue); scale bar: 1 mm. (**B** and **C**) Representative 40× images of the cerebral cortex (**B**) and brain stem (**C**) stained with anti-GM2 ganglioside antibody (red) and counterstained with DAPI (blue); scale bar: 20 μm. (**D**) Quantification of GM2 fluorescence intensity in cortex and brain stem. Small gray symbols represent image-level measurements (technical replicates); large colored symbols indicate per-mouse means (biological replicates). Statistical analysis was performed using a mixed-effects model with Tukey’s correction. ***P < 0.001, ****P < 0.0001. *n* = 3 for WT; *n* = 4 for ABE-treated; *n* = 3 for control-treated. (**E**) Representative 40× images of the cerebral cortex from control- and ABE-treated LOTS mice stained with anti-NeuN (green), anti-GM2 ganglioside (red), and counterstained with DAPI (blue). Merged images (right panels) show colocalization of GM2 and NeuN; scale bar: 20 μm. (**F**) Representative 40× images of the cerebral cortex from control- and ABE-treated LOTS mice stained with anti-LAMP1 (green, pseudocolored) and anti-GM2 ganglioside (red). Merged images (right panels) show colocalization of LAMP1 and GM2; scale bar: 10 μm.

To determine whether base-editor treatment reduced brain GM2 ganglioside levels in LOTS mice, we immunostained brain sections obtained from WT, control-treated LOTS, and ABE-treated LOTS mice with anti-GM2 ganglioside antibody (Figure 4, A–C) and quantified fluorescence intensity in the brain stem and cortex regions (Figure 4D). WT sections showed little staining for GM2 ganglioside. In contrast, control-treated LOTS mice displayed elevated levels of GM2 ganglioside staining, which was significantly reduced in the ABE-treated LOTS mice (Figure 4, A–D).

Costaining with NeuN confirmed that the GM2 ganglioside accumulation in the cortex region of control-treated LOTS mouse brain coincided largely with neurons (Figure 4E). The storage in NeuN-positive neurons was reduced after ABE treatment (Figure 4E). Costaining with LAMP1 indicated that the stored GM2 ganglioside in the cortex region was largely found in intracellular compartments positive for LAMP1, a lysosomal marker (Figure 4F). After ABE treatment, the LAMP-1 compartment along with GM2 ganglioside staining was reduced (Figure 4F).

To quantify the reduction of GM2 ganglioside accumulation by the ABE treatment, we analyzed the ganglioside profiles of ABE-treated and control-treated LOTS brains. High-performance TLC lipid analysis showed that brains from 21-week-old ABE-treated LOTS mice accumulated 11% of the amount of GM2 ganglioside observed in brains from control-treated LOTS mice (Supplemental Figure 5, A and B). The brain GM2 ganglioside level in 21-week-old ABE-treated LOTS was not increased above the level observed in untreated 6.5-week-old LOTS mice, before treatment was initiated (Supplemental Figure 5, C and D).

### Base-editor treatment reduces brain expression of neuroinflammation markers in LOTS mice.

Tay-Sachs disease and other sphingolipid storage disorders exhibit significant neuroinflammation mediated by reactive glia and infiltrating macrophages (24, 25). To evaluate the effect of ABE treatment on neuroinflammation in LOTS mice, we first performed bulk RNA-Seq analysis on brain samples from WT, control-treated, and ABE-treated LOTS mice at 21 weeks of age. Gene Ontology (GO) analysis of the differential gene expression of control-treated LOTS mice relative to ABE-treated LOTS mice showed that the predominant GO categories were related to immune responses, consistent with the expected neuroinflammation (Supplemental Figure 6, A and B). Heatmaps were used to compare the relative gene expression in 5 of the top significant GO categories (leukocyte chemotaxis, myeloid leukocyte activation, leukocyte migration, T cell activation, and regulation of leukocyte activation) among individual mice in the 2 groups (Supplemental Figure 6, C–G). The results indicated a generally increased expression of these immune-related genes in the control-treated LOTS mice compared with ABE-treated LOTS mice.

**Figure 5:**
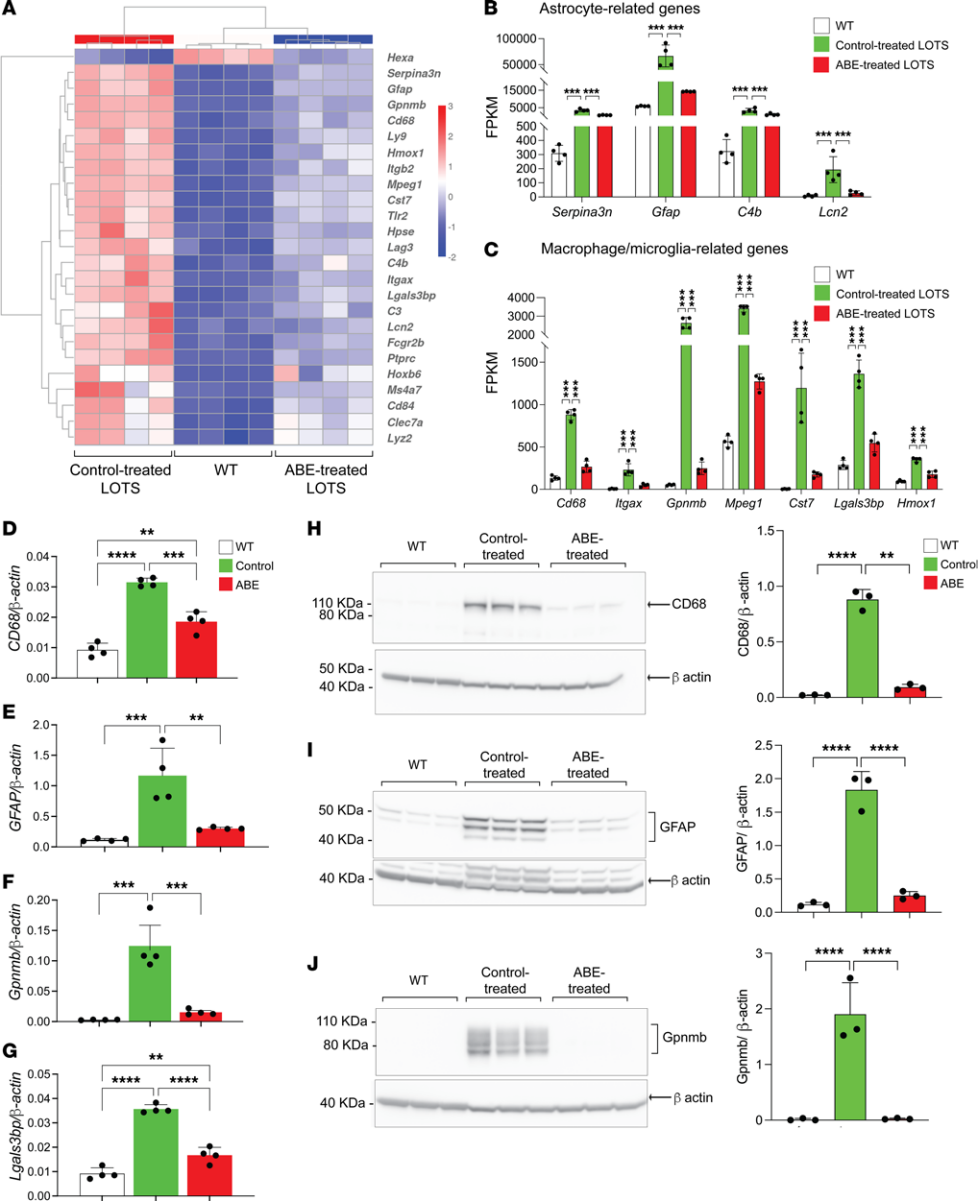
Base-editor treatment reduces brain expression of neuroinflammation markers in LOTS mice. RNA-Seq was performed on brains from WT, control-treated LOTS, and ABE-treated LOTS male mice at 21 weeks of age (*n* = 4 per group). The AAV-treated mice each received 2.4 × 10^12^ vg. Brains were harvested, RNA was extracted, and transcriptome analysis was performed using the NovoMagic platform (Novogene). (**A**) Heatmap showing row *z* scores for the top 25 genes significantly differentially expressed between control-treated LOTS and WT mice. Each column represents an individual mouse. (**B** and **C**) Expression of astrocyte-related genes (**B**) and macrophage/microglia-related genes (**C**) shown as fragments per kilobase of transcript per million mapped reads (FPKM). Data are presented as mean ± SD; each dot represents 1 mouse. ***P < 0.001 (1-way ANOVA with Bonferroni’s correction). (**D**–**G**) Quantitative PCR validation of RNA-Seq results. Expression of *CD68*, *GFAP*, *Gpnmb*, and *Lgals3bp* mRNA in WT, control-treated, and ABE-treated mouse brains. *n* = 4 per group. Data are expressed as mean ± SD. **P < 0.01, ***P < 0.001, ****P < 0.0001 (1-way ANOVA with Bonferroni’s correction). (**H**–**J**) Western blot validation of protein expression. Representative blots (left) and quantification (right) for CD68 (**H**), GFAP (**I**), and Gpnmb (**J**) in brain extracts from WT, control-treated LOTS, and ABE-treated LOTS mice. β-Actin served as a loading control. *n* = 3 per group. Data are expressed as mean ± SD. **P < 0.01, ****P < 0.0001 (1-way ANOVA with Bonferroni’s correction).

The relative expression of the top 25 differentially expressed genes between WT mice and control-treated LOTS mice were examined in WT mice, control-treated LOTS mice, and ABE-treated LOTS mice. We found that ABE treatment modified the expression levels of these genes to be more like those observed in WT mice (Figure 5A). Many of these abnormally expressed genes are linked to glial responses in neuroinflammation, including reactive astrocyte-related genes (*Gfap*, *Serpina3n*, *C4b*, and *Lcn2*) (26) and activated macrophage/microglia-related genes (*Cd68*, *Itgax*, *Gpnmb*, *Mpeg1*, *Cst7*, *Lgals3bp*, and *Hmox1*) (27–29). The base-editor treatment significantly reduced the expression of all these genes in the LOTS mice, indicating a decrease in inflammatory glial and infiltrating macrophage responses (Figure 5, B–J).

**Figure 6:**
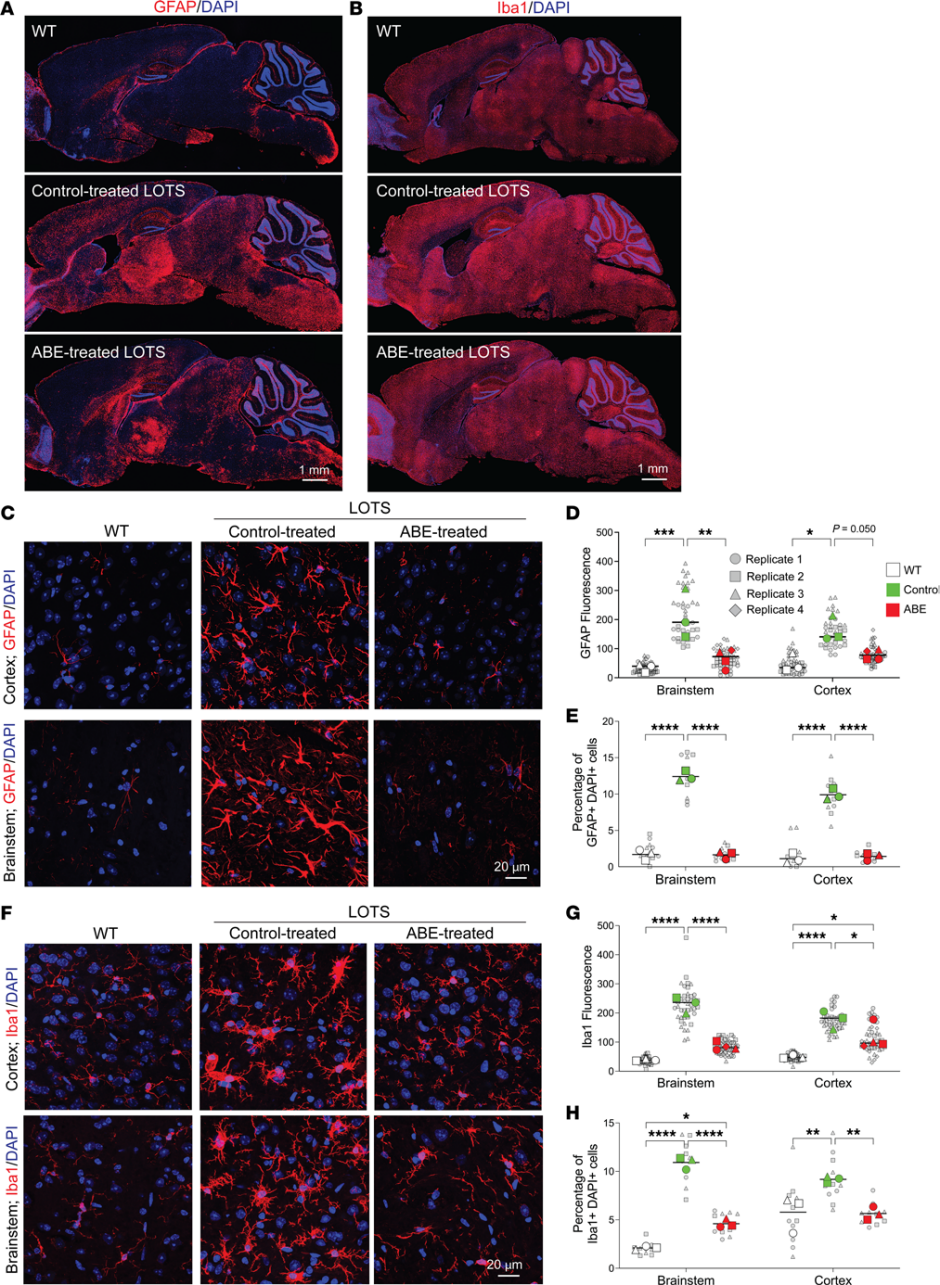
Base-editor treatment reduces glial-cell response in the brain of LOTS mice. WT, control-treated LOTS, and ABE-treated LOTS mice were euthanized at 21 weeks of age, and sagittal brain sections were immunostained (*n* = 4 per group unless otherwise indicated). The AAV-treated mice each received 2.4 × 10^12^ vg. (**A** and **B**) Representative sagittal brain sections stained with anti-GFAP (**A**) or anti-Iba1 (**B**) antibodies (red) and counterstained with DAPI (blue); scale bar: 1 mm (**C** and **F**) Representative 40× images of the cerebral cortex (top panels) and brain stem (bottom panels) stained with anti-GFAP (**C**) or anti-Iba1 (**F**) antibodies (red) and counterstained with DAPI (blue); scale bar: 20 μm. (**D** and **G**) Quantification of GFAP (**D**) and Iba1 (**G**) fluorescence intensity in cortex and brain stem regions. Small gray symbols represent image-level measurements (technical replicates); large colored symbols indicate per-mouse means (biological replicates). Statistical analysis was performed using a mixed-effects model with Tukey’s correction. *P < 0.05, **P < 0.01, ***P < 0.001, ****P < 0.0001. *n* = 3 for WT, *n* = 4 for ABE-treated, *n* = 3 for control-treated. (**E** and **H**) Quantification of activated astrocytes (**E**) and reactive microglia (**H**), expressed as the percentage of GFAP^+^ DAPI^+^ (**E**) or Iba1^+^ DAPI^+^ (**H**) cells relative to total DAPI^+^ nuclei. Cell counts were performed using Fiji software. Small gray symbols represent technical replicates; large colored symbols indicate biological replicates. Statistical significance was assessed using a mixed-effects model with Tukey’s correction. *P < 0.05, **P < 0.01, ****P < 0.0001. *n* = 3 for each group (WT, control-treated, ABE-treated).

We next directly evaluated the effect of base-editor treatment on glial-cell response through immunostaining of brain sections from control-treated LOTS mice and ABE-treated LOTS mice. GFAP immunostaining, identifying astrocytes, revealed reduced signal intensity in the brains of the ABE-treated LOTS mice relative to control-treated LOTS mice (Figure 6, A and C). Quantitative analysis of the GFAP fluorescence signal intensity in the brain stem and cortex confirmed a significant reduction in ABE-treated LOTS mice compared with control-treated LOTS mice, although the GFAP levels in ABE-treated LOTS mice remained significantly increased compared with levels observed in WT mouse brain tissues (Figure 6, C and D). Quantification of GFAP-positive astrocytes demonstrated a significant increase in their numbers in control-treated LOTS mice compared with WT mice (Figure 6E). ABE treatment resulted in a significant reduction in astrocyte numbers in the brain stem and cortex compared with control-treated LOTS mice (Figure 6E).

Iba1 immunostaining, which identifies microglia, was reduced in the brains of ABE-treated LOTS mice compared with control-treated LOTS mice (Figure 6, B and F). Quantitative analysis of Iba1 fluorescence signal intensity in the brain stem and cortex demonstrated findings aligned with those observed for GFAP; that is, Iba1 intensity was significantly reduced in ABE-treated LOTS mice relative to control-treated LOTS mice but remained significantly increased compared with WT mice (Figure 6, F and G). Quantification of Iba1-positive microglia revealed a significant increase in their numbers in control-treated LOTS mice compared with WT mice (Figure 6H). Treatment with ABE led to a marked reduction in microglial numbers in the brain stem and cortex compared with control-treated LOTS mice (Figure 6H).

In control-treated LOTS mice, these glial cells exhibited a large, ramified morphology indicative of an activated state (Figure 6, C and F). After ABE treatment, both astrocytes and microglia displayed a more quiescent morphology, characterized by less ramification and reduced cell body size more similar to glia in the WT cortex. These observations suggest that ABE treatment not only reduces the number and expression levels of reactive glial cells but also promotes a return to a less activated state.

CD68 immunostaining, which identifies both microglia and infiltrating macrophages, also demonstrated an apparent reduction in ABE-treated LOTS brain stem and cortex relative to control-treated LOTS mice (Supplemental Figure 7, A–C). Collectively, these findings are consistent with a robust reactive glial and infiltrating macrophage response occurring in the control-treated LOTS mice, which was substantially reduced after ABE treatment, although not to the level observed in WT mice.

### Base-editor treatment mitigates disease manifestations and prolongs the lifespan of LOTS mice.

**Figure 7:**
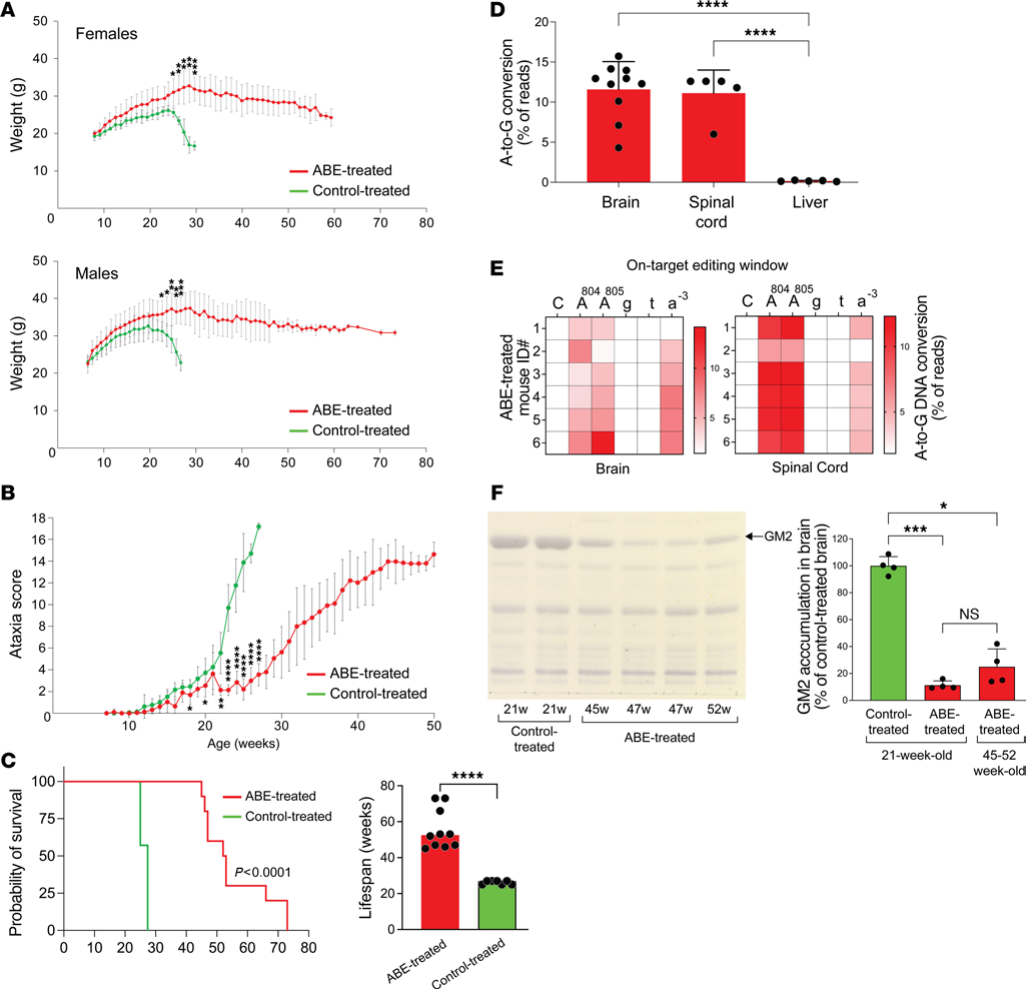
Base-editor treatment mitigates disease manifestations and prolongs the lifespan of LOTS mice. (**A**) Body-weight progression of control- and ABE-treated LOTS mice. The AAV-treated mice each received 2.4 × 10^12^ vg. Mean ± SD shown by sex at each time point (*n* = 6 female and *n* = 4 male ABE-treated; *n* = 3 female and *n* = 4 male control-treated). *P < 0.05, **P < 0.01, ***P < 0.001 (Student’s *t* test). (**B**) Ataxia scores based on 6 behavioral assessments (Figure 2H) collected weekly after treatment until death. Data shown as mean ± SD (*n* = 34 ABE-treated, *n* = 32 control-treated; includes all mice in Supplemental Table 2). *P < 0.05, **P < 0.01, ****P < 0.0001 (Student’s *t* test). (**C**) Left: Kaplan-Meier survival plot for ABE- and control-treated LOTS mice (*n* = 10 and *n* = 7, respectively). *P* < 0.0001 (log-rank test). Right: median survival of each group. Each dot represents 1 mouse. ****P < 0.0001 (1-way ANOVA with Bonferroni’s correction). (**D**) On-target editing efficiency at HEXA c.805A site in brain, spinal cord, and liver DNA from end-stage ABE-treated LOTS mice (age 45–52 weeks). A-to-G conversion shown as mean ± SD from next-generation sequencing (*n* = 10 brain; *n* = 5 spinal cord and liver). ****P < 0.0001 (1-way ANOVA). (**E**) Heatmaps of A-to-G editing at the on-target locus in brain (right) and spinal cord (left) of individual end-stage ABE-treated mice. Protospacer sequence shown above; uppercase = exon, lowercase = intron. (**F**) Brain GM2 ganglioside levels in control-treated (21 weeks), ABE-treated (21 weeks), and end-stage ABE-treated LOTS mice. Left: representative high-performance TLC plate of gangliosides (0.5% of hemisphere extract). Arrow marks GM2 standard. Right: quantification of GM2 levels relative to control (set at 100%). Mean ± SD (*n* = 4/group). *P < 0.05, ****P < 0.0001; ns = not significant (ANOVA with Bonferroni’s correction).

We monitored ABE-treated and control-treated LOTS mice (both males and females) throughout their lifespans. Each mouse underwent weekly evaluations, including body-weight measurements and a series of 6 tests to calculate an ataxia score (Figure 2H). Starting around week 22, control-treated LOTS mice began to show a sharp decrease in body weight. In contrast, ABE-treated LOTS mice maintained their weight gain until about week 30, followed by a gradual decline; however, their weight never fell below 75% of the maximum value (Figure 7A and Supplemental Figure 8).

Control-treated LOTS mice began to exhibit ataxia symptoms after week 10, with the maximum ataxia score being reached by week 27 just prior to their end of life. ABE-treated LOTS mice initially showed mild ataxic symptoms after 14 weeks of age. After about 21 weeks, these symptoms slowly worsened over time but were significantly better than the control-treated LOTS mice (Figure 7B and Supplemental Videos 1 and 2). Their maximum ataxia score was reached by approximately week 50. Control-treated LOTS mice had a median lifespan of around 27 weeks, whereas ABE-treated LOTS mice demonstrated a significantly extended median lifespan of 52.5 weeks (Figure 7C). These results demonstrated that base-editor treatment both delayed disease symptom onset and substantially extended the lifespan of LOTS mice.

The editing of the *HEXA* c.805G>A mutation in the brain, spinal cord, and liver was similar in ABE-treated LOTS mice that survived to 45 to 52 weeks and ABE-treated LOTS mice at 21 weeks of age, both in the frequency of the mutation correction and bystander editing (Figure 7, D and E, and Figure 3, B–D). Finally, we compared the level of GM2 ganglioside accumulation in the brains of ABE-treated LOTS mice that survived to 45 to 52 weeks to control-treated and ABE-treated LOTS mice that were euthanized at 21 weeks. The level of GM2 ganglioside in the older ABE-treated LOTS mice was only about 25% of the level observed in control-treated LOTS mice at 21 weeks. Notably, this level was not significantly different from the accumulation of GM2 observed in the ABE-treated LOTS mice at 21 weeks, indicating that base-editor treatment effectively sustained the prevention of progressive GM2 ganglioside accumulation in the brain over time (Figure 7F).

## Discussion

The pathogenic *HEXA* point mutation, c.805G>A, is carried by most patients with LOTS, either in homozygous form or in combination with a different *HEXA* mutation (6–9). This c.805G>A mutation leads to a serine-to-glycine substitution at position 269 in the α subunit of lysosomal β-hexosaminidase A, reducing enzyme activity below the threshold needed for sufficient degradation of GM2 ganglioside. LOTS typically manifests in young adults and is characterized by a gradually progressing range of neurological symptoms that can include ataxia, weakness, spasticity, dysarthria, peripheral neuropathy, psychosis, and cognitive decline (5). Currently, no cure for LOTS exists and treatment options are limited (1). In this study, we explored the therapeutic potential of base editing to permanently correct the common LOTS mutation in the CNS.

Here, we first tested base editing in fibroblasts obtained from a patient with LOTS who was homozygous for the c.805 G>A mutation. Adenine base editing partially corrected the mutation and partially restored enzymatic activity mediated by the α subunit of β-hexosaminidase A, with minimal genome-wide off-target editing. To assess the efficacy of this approach in the CNS, we developed a LOTS mouse model that carried the *HEXA* c.805G>A point mutation and exhibited major features of the disease: low α subunit–specific β-hexosaminidase activity; GM2 ganglioside accumulation in the brain; and progressively worsening neurological manifestations. These mice also exhibited a substantially reduced lifespan, which reflects a more severe disease than what is observed in humans with LOTS. Despite this severe disease course, a one-time intravenous administration of AAV carrying the ABE and LOTS-sgRNA to adult LOTS mice resulted in a substantial improvement of disease features. β-Hexosaminidase activity was partially restored, brain GM2 ganglioside accumulation was reduced, and the upregulation of neuroinflammation markers in the brain typically seen in sphingolipid storage diseases was mitigated. Moreover, this treatment delayed the emergence of severe symptoms and significantly extended the median lifespan of the mice, demonstrating the potential of base editing as a treatment for LOTS associated with the *HEXA* c.805G>A mutation.

In the brain and spinal cord of LOTS mice, ABE treatment corrected the c.805 G>A mutation with a frequency of approximately 10%, leading to a commensurate increase in brain α subunit–specific β-hexosaminidase activity. Notably, GM2 ganglioside storage in the brain was reduced nearly 90% in ABE-treated LOTS mice, which is much greater than what might be expected based on the mutation correction frequency alone. This finding suggests that the benefits of the mutation correction extend beyond the directly edited cells. This disproportionally large impact on GM2 ganglioside accumulation could be explained by cross-correction (30). In this scenario, active β-hexosaminidase A produced in cells where the mutation has been successfully corrected could be secreted and then taken up by cells that have not been edited. β-Hexosaminidase A carries the mannose-6-phosphate recognition marker (31, 32), which directs it to lysosomes through cell-surface mannose-6-phosphate receptors, providing a potential mechanism for the delivery to unedited cells. Further investigation is needed to determine the extent of correction required to fully alleviate disease manifestations and to confirm the cross-correction mechanism.

Recent clinical trials of gene therapy for glycosphingolipid storage diseases have employed AAV vectors (33). The viral vectors have been delivered both systemically and directly into the CNS via combined thalamic and cerebrospinal fluid delivery. Gene therapy for infantile Tay-Sachs disease has involved the use of 2 monocistronic AAV vectors separately encoding the *HEXA* and *HEXB* genes (18). The dual-vector approach was necessary to ensure balanced cellular synthesis of the α and β subunits for optimal production of the β-hexosaminidase A heterodimer. These trials provided evidence of safety, but treatment efficacy has not been reported. Base-editing therapies can in principle be delivered either systemically or directly into the CNS depending on the extent of disease manifestations. A key advantage of base editing is its potential for a one-time, permanent correction of the mutant *HEXA* allele, eliminating the need for repeated dosing of vectors and any potential immunological complications. Moreover, mutation correction would also result in the normal synthesis levels of the α subunit under control of the endogenous *HEXA* promoter. The result would be optimal levels of α subunit for heterodimer formation with the β subunit for production of β-hexosaminidase A.

When ABE-treated LOTS mice ultimately succumbed to their disease, analysis of their brain tissue indicated that the reduction of GM2 ganglioside levels was to a degree that would be sustainable for life. This suggests that the base editing might not have effectively reached certain critical regions in the CNS or peripheral tissues that require β-hexosaminidase A activity to support a normal lifespan. Mutation correction analysis across brain regions showed that the cerebellum had the lowest level of correction. Additionally, we observed minimal editing in the sciatic nerve of ABE-treated mice, consistent with the known tropism of PHP.eB (20). Thus, inadequate mutation correction in both the cerebellum and PNS, tissues affected in patients with LOTS (5), may have contributed to the eventual decline of ABE-treated mice.

Translating base-editing therapy for LOTS into clinical application presents significant challenges. Adults often possess preexisting neutralizing antibodies against AAV capsids, which can impede viral transduction and reduce therapeutic efficacy. Additionally, high doses of AAV vectors for effective intravenous delivery in adults could raise concerns regarding cost and potential adverse reactions, including immune responses to both the AAV capsid and the ABE. Lower therapeutic doses may be attained by packaging the ABE into a single AAV, eliminating the need for dual-vector delivery. Recent advancements in compact ABEs have demonstrated efficient editing at comparable or even lower doses than the dual-AAV strategy (34, 35). Given the critical role of microglia in GM2 gangliosidosis, particularly in supplying neurons with β-hexosaminidase, enhancing AAV expression in microglia could improve editing efficiency and directly target a key cell type involved in disease pathogenesis (36, 37). Finally, because PhP.eB, the AAV used in this study, does not cross the blood-brain barrier in humans, alternative AAV serotypes capable of achieving widespread nervous system distribution in humans will be required (38).

Lysosomal storage diseases typically manifest along a spectrum, characterized by variable severity and age of onset, and are often categorized into infantile, juvenile, and late-onset or adult forms (39). In the most severe infantile forms, pathology begins in utero and rapidly progresses postnatally. Ideally, to curtail the progression of pathology, base-editing therapy would begin soon after birth or even prenatally (40). In contrast, juvenile and adult forms have a broader time window before the onset of significant pathology and disease manifestations, allowing for more flexibility for when to initiate base-editing therapy. LOTS, in particular, is frequently subject to misdiagnosis or delayed diagnosis (4). Accurate and early diagnosis will be important for LOTS to ensure that potential base-editing treatments can be initiated early enough to effectively block disease progression.

With its various clinical forms, Tay-Sachs disease is a prototype within the family of sphingolipid storage diseases characterized by single lysosomal enzyme deficiencies, a group that includes GM1 gangliosidosis, as well as Sandhoff, Niemann-Pick, Krabbe, and Gaucher diseases, and others (39). These disorders typically are characterized by progressive neurodegeneration. While some of these diseases exclusively affect the nervous system, others also present with peripheral symptoms. For several of these diseases, common pathogenic mutations have been identified, providing therapeutic targets for subsets of patients. Correcting the underlying genetic cause of the diseases by base editing opens the possibility for one-time, permanent treatments potentially halting the progression of these devastating disorders.

## Methods

### Sex as a biological variable.

Our study examined male and female mice, and similar findings are reported for both sexes.

### Cell culture.

Human fibroblasts were grown in DMEM (Thermo Fisher Scientific) supplemented with 10% FBS (HyClone Laboratories, GE Healthcare Life Sciences).

### β-Hexosaminidase assay.

Lysates of human fibroblasts or mouse tissues were prepared in 0.1 M citrate buffer pH 4.2 containing 0.1% Triton X-100 and assayed for β-hexosaminidase A activity with 4-methylumbelliferyl-6-sulfo-N-acetyl-β-D-glucosaminide (Sigma-Aldrich) (41). Activity was calculated as β-hexosaminidase activity per minute normalized by protein concentration, determined using the Pierce BCA assay (Thermo Fisher Scientific).

### Genomic DNA preparation for sequencing.

Genomic DNA was prepared from brain, liver, spinal cord, and skin fibroblasts using the DNAeasy Blood & Tissue Kit (QIAGEN).

### Mouse generation and genotyping.

*HEXA* mouse lines were generated by CRISPR/Cas9 genome editing as described (42–44). Briefly, 2 sgRNAs targeting intron sequences flanking exon 7 of the mouse *Hexa* gene (Hexa_EX7_sg2 GACGAGTGTTCTAAAATTCA and Hexa_EX7_sg3 CTAGAGGGAGTTCCTTCCAT; designed using the University of California Santa Cruz Genome Browser and synthesized by Synthego) and a donor template (ssDNA [synthesized by Integrated DNA Technologies] oligonucleotides containing the *HEXA* exon 7, flanking introns, and mouse homology arms) were injected into 1-cell mouse C57BL/6J embryos. The donor templates were identical except for position *HEXA* c.805, which carried the reference G (for ssDNA oligo *HEXA* exon 7 c.805G) or the mutant A (for ssDNA oligo *HEXA* exon 7 c.805A) (6).

Mouse offspring were screened by PCR, using tail-snip DNA to identify a founder mouse carrying the human sequences, with the following primers and conditions: HexA_HuF2: 5′ CTCTGCTAGCTTTCAGGAAGTGTG 3′ and HexA_HuR2: 5′ TAAGGACCAAGGCTGGGATATGC 3′ with the following PCR conditions: denaturation, 95°C for 5 minutes; amplification, 95°C for 30 seconds, 61°C for 15 seconds, 68°C for 30 seconds; and extension, 72°C for 7 minutes (40 cycles). The expected product size is 288 bp. The amplified fragments were sequenced to confirm orientation of the knock-in fragment and the entire inserted sequence using the following primers and PCR conditions, followed by Sanger sequencing: MuHexa_F1 5′ ACCTCCTAATAGTTCCACTCTCT 3′ and HexA_HuR2 5′ TAAGGACCAAGGCTGGGATATGC 3′; the expected product size is 593 bp. HexA_HuF2 5′ CTCTGCTAGCTTTCAGGAAGTGTG 3′ and MuHexa_R1 5′ GCAACCAGTTCTCTTAATTGTTGA 3′; the expected product size is 544 bp. SeqPrimer1F 5′ GCTTCGGACGAGTGTTCTAAA 3′ and SeqPrimer1R 5′ CTTGAGCCACTTTCTTGCTTTAC 3′; the expected product size is 423 bp. SeqPrimer2F 5′ CAGAGGGACTCTGCTTGTTATG 3′ and SeqPrimer1R 5′ CTTGAGCCACTTTCTTGCTTTAC 3′; the expected product size is 162 bp. The following were the PCR conditions: denaturation, 94°C for 5 minutes; amplification, 94°C for 30 seconds, 62°C for 15 seconds, 68°C for 30 seconds; and extension, 72°C for 7 minutes (40 cycles).

*HEXA* c.805A mice were genotyped by PCR of tail-snip DNA, using the following primers and PCR conditions: HexA_mou_For1 5′ AGGAACACACACAATGGTGCT 3′, HexA_MuHuR2 5′ TAAGGACCAAGGCTGGGATATGC 3′, and Hexa KI HoHetR1 5′ CCCTCTTTTAGCAGACGCCTC 3′ with the following PCR conditions: denaturation, 95°C for 10 minutes; amplification, 95°C for 30 seconds, 62°C for 30 seconds, 72°C for 1 minute; and extension, 72°C for 7 minutes (40 cycles). The expected product size for the WT mouse *Hexa* allele is 409 bp and for the humanized *HEXA* c.805A allele is 470 bp.

*HEXA* c.805G mice were genotyped by PCR of tail-snip DNA, combining 2 PCR reactions using the following primers and PCR conditions: for PCR1, HEXA MuHuF2: 5′ CTCTGCTAGCTTTCAGGAAGTGTG 3′ and HEXA MuHuR2: 5′ TAAGGACCAAGGCTGGGATATGC 3′ with the following PCR conditions: denaturation, 95°C for 10 minutes; amplification, 95°C for 30 seconds, 61°C for 15 seconds, 68°C for 30 seconds; and extension, 72°C for 7 minutes (40 cycles); the expected product size for the humanized *HEXA* c.805G allele is 288 bp. For PCR2: HexA MuF1: 5′ AGTTGTAGAGGAACACACACAATGG 3′ and Hexa KI HoHetR1 5′ CCCTCTTTTAGCAGACGCCTC 3′ with the following PCR conditions: denaturation, 95°C for 10 minutes; amplification, 95°C for 30 seconds, 61°C for 15 seconds, 68°C for 30 seconds; and extension, 72°C for 7 minutes (40 cycles); the expected product size for the WT mouse *Hexa* allele is 417 bp.

*Neu3-*KO mice were generated by CRISPR/Cas9-targeted gene disruption as described (43). *Neu3-*KO mice were genotyped by PCR of tail-snip DNA, using the following primers and PCR conditions: Neu3_For1 5′ CTAGAGAACAGAGTTGTTGCATGAGG 3′, Neu3_Rev13 5′ GAGGCCTGTAGCAGTGAATTAGTTAAAC 3′, and Neu3_Rev5 5′ GCTAGTTGGATGTGAGTACAAGAG 3′ with the following PCR conditions: denaturation, 94°C for 10 minutes; amplification, 94°C for 30 seconds, 63°C for 30 seconds, 72°C for 1 minute; and extension, 72°C for 7 minutes (40 cycles). The expected product size for the mouse *Neu3* WT allele is 502 bp and for the mouse *Neu3-*KO allele is 328 bp.

*HEXA* c.805A and *HEXA* c.805G mice were crossed with *Neu3-*KO mice to generate the *HEXA* c.805A/*Neu3-*KO LOTS mice and *HEXA* c.805G/*Neu3-*KO mice, respectively. C57BL/6J mice (The Jackson Laboratory) were used as controls.

### ABEs.

Lentiviruses were used for base-editor treatment of patient-derived skin fibroblasts. Two separate lentiviruses were used. The base editor, ABE7.10 RA, was carried by pLenti-ABERA-P2A-Puro, a gift from Lukas Dow (Weill Cornell Medicine, New York, New York, USA) (Addgene plasmid 112675) (45). The LOTS-sgRNA 5′ GACCAAGTAAGAATGATGTC 3′ was cloned into pLenti coexpressing EGFP (Genscript). Lentiviruses were packaged using Lenti-X Packaging Single Shots (VSV-G) (631276, Takara Bio) following the manufacturer’s protocol. Lentiviruses were concentrated using Lenti-X Concentrator (631232, Takara Bio), followed by titer determination using Lenti-X GoStix Plus (631281, Takara Bio) following the manufacturer’s protocols.

For base editing in mice, a dual AAV system was used to accommodate the ABE (v5 AAV-ABE) and the LOTS-sgRNA. The v5 AAV-ABE was divided into N-terminal and C-terminal segments, equipped with split-intein fragments, and expressed from the Cbh promoter. Cbh_v5 AAV-ABE N-terminal (Addgene plasmid 137177) and Cbh_v5 AAV-ABE C-terminal (Addgene plasmid 137178) plasmids were gifts from David Liu (Harvard University, Cambridge, Massachusetts, USA) (21). The LOTS-sgRNA was incorporated into the Cbh_v5 AAV-ABE C-terminal plasmid. The 2 plasmids were packaged into AAV-PHP.eB (VectorBuilder). pAAV[Exp]-CMV>EGFP:WPRE packaged into AAV-PHP.eB (VectorBuilder) was used as a control virus.

### Lentivirus transduction of human fibroblasts.

Fibroblasts from a patient with LOTS were plated at 100,000 cells/well in a 24-well plate to achieve 50%–70% confluence. pLenti-ABERA-P2A-Puro lentivirus carrying the base editor was added at an MOI of 5. Puromycin (Thermo Fisher Scientific) was added 48 hours after transduction at 1 μg/mL to select for infected cells and was maintained throughout the cultures. Puromycin-resistant fibroblasts were transduced using lentivirus expressing the LOTS-sgRNA (MOI = 5), and double-transduced fibroblasts were selected using flow cytometry, based on the coexpression of EGFP.

### Disease assessment.

Mice were weighed weekly starting at 6 weeks of age. Ataxia scoring was adapted from Guyenet et al. (46) and applied to determine disease severity. Briefly, each animal was subjected weekly to a set of 6 assessments, which were recorded on a scale of 0–3 (0 represented absence of the phenotype and 3 represented the most severe manifestation). The ataxia score was produced by summing the 6 assessment values for a single mouse. The assessments used were the ledge test to measure coordination; hindlimb clasping; gait to test coordination and muscle function; kyphosis as a manifestation of neurodegeneration; stance; and hindlimb locomotion (46).

### Retro-orbital injections of AAV.

Vascular delivery of AAV-PHP.eB (a total dose of 2.4 × 10^12^ vg/mouse) was performed by retro-orbital injection (47). Mice at 6–7 weeks old were anesthetized using isoflurane, and virus was injected into the retrobulbar sinus of the right eye using a 29-gauge, 0.5 mL tuberculin syringe.

### Next-generation sequencing of LOTS mutation.

Genomic DNA was amplified using primers flanking *HEXA* c.805 on exon 7 to introduce partial Illumina adapter sequences: PA_For 5′ ACACTCTTTCCCTACACGACGCTCTTCCGATCTGGTATCCGTGTGCTTGCAGAG 3′ and PA_Rev 5′ GACTGGAGTTCAGACGTGTGCTCTTCCGATCTTAAGGACCAAGGCTGGGATATGC 3′ with the following PCR conditions: denaturation, 98°C for 10 minutes; amplification, 98°C for 10 seconds, 65°C for 30 seconds, 72°C for 30 seconds; and extension, 72°C for 7 minutes (27 cycles). The resulting PCR product (226 bp) was gel-purified and subjected to Amplicon-EZ next-generation sequencing (GeneWiz, Azenta Life Science).

FASTQ files were analyzed using CRISPRESSO2 (48) to determine the percentage of conversion of A•T to G•C at the point-mutation site. Each sample had approximately 120,000 to 180,000 total reads.

### RNA-Seq analysis.

Brains from *HEXA* exon 7 c.805G *Neu3-*KO mice (both ABE-treated and control-treated) and from WT mice were harvested at 21 weeks of age. RNA-Seq analysis was performed as described (42, 43). Briefly, total RNA from 1 brain hemisphere was isolated using the miRNeasy Mini Kit from QIAGEN (217004). Preparation of the RNA library, mRNA-Seq, and bioinformatic analysis were performed by Novogene. Libraries were sequenced on an Illumina PE150 platform for 40 million paired-end reads for each sample. Data were analyzed using the cloud platform NovoMagic (Novogene).

### Immunohistology and histochemistry.

Mouse brains were fixed in paraformaldehyde (4% in PBS), placed in 20% sucrose overnight until the tissue sank, embedded in OCT compound (4583, Sakura Finetek), and frozen over dry ice. Frozen brain blocks were sectioned sagittally using a cryostat (CM1950, Leica Biosystems). Sections were mounted on glass slides, washed in PBS to remove the OCT compound, and air-dried. For GM2 ganglioside immunostaining only, sections were permeabilized with prechilled (–20°C) acetone for 10 minutes at –20°C and air-dried prior to blocking/antibody incubations. All sections were incubated with Mouse on Mouse blocking reagent (MKB-2213-1, Vector Laboratories).

For GM2 ganglioside immunostaining, sections were incubated with 10% normal goat serum (Thermo Fisher Scientific) in PBS, and then incubated with anti-GM2 ganglioside antibody (IgM mouse monoclonal; A2576, TCI) diluted in 2% normal goat serum in PBS for 1 hour at room temperature.

For other primary antibodies, sections were incubated with 10% normal goat serum with 0.3% Triton X-100 in PBS for 1 hour at room temperature. Sections were then incubated overnight at 4°C with anti-GFAP (IgG, rabbit polyclonal, ab7260, Abcam), anti-Iba1 (IgG, rabbit polyclonal, 019-19741, Wako), anti-CD68 (IgG, rabbit monoclonal E307V, 97778, Cell Signaling Technology), or anti-Lamp1 (rat monoclonal, MABC39, MilliporeSigma), diluted in 2% normal goat serum/PBS.

After 3 washes in PBS, sections were incubated for 1 hour at room temperature with the following secondary antibodies (all from Thermo Fisher Scientific) diluted 1:400 in 2% normal goat serum in PBS: goat anti-mouse IgM (heavy chain) Alexa Fluor 594 (A-21044); goat anti-rabbit IgG (H+L) DyLight 594 (35561); or goat anti-rat IgG (H+L) Alexa Fluor Plus 405 (A48261). Sections were washed and counterstained with DAPI (Thermo Fisher Scientific), mounted with Prolong Diamond Antifade Mountant (P36961, Thermo Fisher Scientific) and, after curing for 24 hours, imaged under a Zeiss confocal microscope. For sections stained with multiple fluorescent probes, images of different channels were analyzed and merged using Fiji/ImageJ (NIH) to determine relationships between different labels. For fluorescence intensity studies, 3–5 *Z*-stacks from each sample were captured under 40× or 63× oil immersion objectives with the same settings. With 10 to 15 images/stack, images were analyzed for fluorescence intensity per pixel using Fiji/ImageJ.

### Statistics.

Depending on the dataset, statistical significance was assessed using 2-tailed Student’s *t* test, 1-way ANOVA with Bonferroni’s correction, 2-way ANOVA with Bonferroni’s correction, mixed-effects model with Tukey’s correction, or log-rank (Mantel-Cox) test for survival analysis. *P* values less than or equal to 0.05 were considered statistically significant.

### Study approval.

LOTS fibroblasts were derived from a skin biopsy in a patient with LOTS. The LOTS donor was an adult male patient homozygous for the *HEXA* c.805G>A mutation. Control human primary fibroblasts were established from a skin biopsy of an unaffected donor. The tissue samples included in this study were from patients enrolled in an NIH protocol, “Natural History of Glycosphingolipid Storage Disorders and Glycoprotein Disorders” (IRB 09-HG-0107, NCT00029965), which was approved by the National Human Genome Research Institute IRB. Written informed consent was received from participants prior to inclusion in the study. All studies involving human participants abided by the Declaration of Helsinki principles. All animal procedures were approved by the National Institute of Diabetes and Digestive and Kidney Diseases IACUC and were performed in accordance with NIH guidelines.

### Data availability.

RNA-Seq data presented in this report are accessible in the NCBI’s Gene Expression Omnibus (GEO GSE268712). Values for all data points in graphs are reported in the Supporting Data Values file. All other raw data analyzed and described in this report are available from the corresponding author upon reasonable request. Further information can be found in the Supplemental Methods.

## Author contributions

RLP and MLA designed the study. MLA, MK, YTL, SMO, VH, JYB, FP, CL, CB, GT, and HZ conducted experiments. MLA, MK, YTL, SMO, VH, JYB, CL, CB, GT, HZ, CJT, and RLP analyzed the data. CJT provided patient samples. RLP and MLA wrote the manuscript. MLA, MK, YTL, SMO, VH, JYB, FP, CL, CB, GT, HZ, CJT, and RLP reviewed and edited the manuscript.

## Supplementary Material

Supplemental data

Unedited blot and gel images

Supplemental video 1

Supplemental video 2

Supporting data values

## Version 1 06/17/2025

In-Press Preview

## Version 2 08/15/2025

Electronic publication
