# Supplementary material for: CNS-targeted base editing of the major late-onset Tay-Sachs mutation alleviates disease in mice
Source: J Clin Invest. 2025 Jun 17;135(16):e183434. doi: 10.1172/JCI183434 (PMC12352896; doi:10.1172/JCI183434)
Supplement: Supplemental data [file jci-135-183434-s103.pdf]

Supplemental Material

CNS-Targeted Base Editing of the Major Late-Onset Tay-Sachs Mutation Alleviates Disease in Mice

Maria L. Allende[1], Mari Kono[1], Y. Terry Lee[1], Samantha M. Olmsted[1], Vienna Huso[1], Jenna Y. Bakir[1], Florencia Pratto[1], Cuiling Li[1], Colleen Byrnes[1], Galina Tuymetova[1], Hongling Zhu[1], Cynthia J. Tifft[2], and Richard L. Proia[1]

[1]Genetics and Biochemistry Branch, National Institute of Diabetes and Digestive and Kidney Diseases, NIH

[2]Medical Genetics Branch, National Human Genome Research Institute, NIH

Supplemental Methods

Off-target analysis by CIRCLE-seq. Off-target analysis of the AAV.ABE editor targeting *HEXA* c.805G>A was performed using CIRCLE-seq. CIRCLE-seq was performed as described (1, 2), with modifications. Initially, 100 µg of human genomic DNA (catalog number 11691112001, Roche, MilliporeSigma) was sheared to an average length of 300 bp using a Covaris M220 Focused-Ultrasonicator instrument following standard parameters. The sheared genomic DNA was divided into 2 samples, and each was individually prepared into a CIRCLE-seq library by undergoing end repair, poly-A tailing, and uracil-containing stem-loop adapter ligation utilizing the KAPA HyperPrep Kit (catalog number KK8500, Roche Sequencing). Next, the adapter-ligated DNA was treated with Lambda exonuclease (catalog number M0262S, New England Biolabs) and *Escherichia coli* exonuclease I (catalog number M0293S, New England Biolabs) and then treated with USER enzyme (catalog number M5505S, New England Biolabs) and T4 polynucleotide kinase (catalog number M0201S, New England Biolabs). The treated DNA was then intermolecularly circularized by the T4 DNA ligase enzyme (catalog number M0202S, New England Biolabs) and cleared of any residual linear DNA by Plasmid-safe ATP-dependent DNase (catalog number E3101K, Biosearch Technologies). In vitro cleavage reactions utilized *Streptococcus pyogenes* Cas9 nuclease (catalog number M0386T, New England Biolabs) and LOTS-sgRNA (Synthego) containing a phosphonothioate linkage and 2'MeO modification at the first and last 3 nucleotides to form Cas9:LOTS-sgRNA ribonucleoprotein complexes. The Cas9:LOTS-sgRNA cleavage mix was added to 2 samples of 125 ng of circularized DNA. Two negative control samples with 125 ng of circularized DNA without

Cas9:LOTS-sgRNA were also included. The 2 cleaved DNA and 2 negative control samples underwent next-generation sequencing library preparation by poly-A tailing (Klenow fragment 3’-5’ exo, catalog number M0212S, New England Biolabs), NEBNext Adapter for Illumina ligation (included in the NEBNext Multiplex Oligos for Illumina kit, catalog number E7600S, New England Biolabs), and treatment with USER enzyme (included in the NEBNext Multiplex Oligos for Illumina kit). Finally, a PCR reaction set up with 20 ng of A-tailed, USER enzyme-treated DNA, and the NEBNext i5 and i7 primers (included in the NEBNext Multiplex Oligos for Illumina kit) was run with unique dual-index barcode sequence combinations for each sample. Following Qubit 1x dsDNA high-sensitivity assay (catalog number Q33230, Invitrogen) quantification, the samples were subjected to next-generation sequencing by Illumina MiSeq (Admera Health). The resulting data were analyzed using CIRCLE-seq software (https://github.com/tsailabSJ/circleseq) with the default recommended parameters and the human reference genome GRCh37 for alignment. This analysis identified off-target double-stranded breaks from Cas9:LOTS-sgRNA throughout the entire human genome. From the list of the top 25 identified off-target loci with the greatest read counts for each sample, 18 loci were shared by both human genomic samples. To determine the possible base editing within the sequence corresponding to those 18 loci in ABE-treated LOTS fibroblasts as the result of off-target effects, PCR primers were designed to amplify all 18 sites. PCR products were successfully obtained for 11 off-target sites (identified with an * in Supplemental Figure 1A and listed in the top portion of Supplemental Table 1). The on-target site on *HEXA* was included as a positive control for base editing in ABE-treated LOTS fibroblasts. Primers and amplicon genomic

positions for the 18 identified off-target locus are listed in Supplemental Table 1. The PCR products were about 150 and 500 bp, not including the partial Illumina adapter sequences. The adapter for the forward sequence is 5' ACACTCTTTCCCTACACGACGCTCTTCCGATCT 3' and the adapter for the reverse sequence is 5' GACTGGAGTTCAGACGTGTGCTCTTCCGATCT 3'. The PCR conditions were denaturing at 95° C for 5 min, 29 amplification cycles of 98° C for 10 sec, 65° C for 30 s, and 72° C for 30 s, and a final extension at 72° C for 7 min, followed by a hold at 4° C. The PCR products obtained using genomic DNA from ABE-treated LOTS fibroblasts and from untreated LOTS fibroblasts were sequenced using Amplicon-EZ next-generation sequencing (GeneWiz).

Microglia editing. Microglia were isolated from 26-week-old ABE-treated and 25-week-old untreated LOTS mice using the CD11b Microbeads, human and mouse kit (catalog number 130-097-142, Miltenyi Biotec) as described (3). Genomic DNA from whole brain (input), flow through and CD11b-enriched cells (CD11b^{+}) was obtained and subjected to amplicon deep sequencing to determine LOTS mutation correction.

Real-time quantitative PCR. Total RNA was isolated from both ABE-treated and control-treated LOTS and from WT mice brains using miRNease Mini Kit (catalogue #217004, Qiagen). 2 mg of total RNA were reversed transcribed into cDNA using SuperScript IV VILO Master Mix (catalogue #11756050, Thermo Fisher Scientific). The real-time quantitative PCR was conducted with 10 ng of cDNA from each genotype using Quant Studio 7 with TaqMan Gene Expression Master Mix (catalogue #4369016, Thermo

Fisher Scientific). TaqMan probes for mouse *GFAP* (Mm01253033_m1), mouse *CD68* (Mm03047343_m1), mouse *Gpnmb* (Mm01328587_m1), *Lgals3bp* (Mm00478303_m1) and β–actin (*Actb*, Mn00607939S1) were purchased from Thermo Fisher Scientific. Relative gene expression analysis was performed with β-actin as endogenous control.

Proximity Ligation Assay (PLA). Full-length ABE expression was assessed using the Duolink® In Situ Red Starter Kit ((Millipore Sigma) on frozen sections of brain from 21-week-old ABE-treated and control-treated LOTS mice. To detect reconstituted full-length ABE, we employed two antibodies targeting distinct termini of Cas9: a mouse monoclonal anti-Cas9 antibody specific to the N-terminus (clone 7A9, catalog number MAC133, Millipore Sigma) and a rabbit monoclonal anti-Cas9 antibody specific to the C-terminus (clone EPR18991, catalog number ab189380, Abcam). Imaging was performed using a Zeiss confocal microscope. For quantification, three Z-stacks were acquired from each brain region (cortex, cerebellum, thalamus, midbrain, pons, and medulla) at 40× magnification, with 10–15 images collected per stack. A positive PLA signal—indicating that both antibodies were within <40 nm of each other—confirmed reconstitution of the full-length ABE. Cells were considered positive for full-length ABE expression when two or more red fluorescent PLA clusters were observed adjacent to a DAPI-positive nucleus. Positive cells were manually counted.

Sphingolipid purification and analysis. Glycosphingolipid analysis of mouse brain was performed by high-performance TLC (HPTLC). Total lipids were extracted from 1 mouse hemisphere using 10 vol (v; v/wt) of chloroform:methanol (C:M) (1:1 v/v), followed by 10

vol of C:M (1:2 v/v) and by 10 vol of C:M:H_2O (30:60:8 v/v/v). Each lipid extraction was performed for 16 h at room temperature. The combined lipid extracts were dried under N_2, dissolved in 9 mL C:M (2:1), and partitioned into 2 phases after the addition of 0.2 vol of 0.1 M KCl (4).

Acidic glycosphingolipids, mainly gangliosides, were isolated from the upper (aqueous) phase. The upper phase was desalinated using a Sep-Pak C18 column (part number WAT020805; Waters) that was previously washed with 3 column vol of M, 5 column vol of M:H_2O (1:1 v/v), and 2 column vol of 0.1 M KCl. After sample application, columns were washed with 5 column vol of H_2O and gangliosides were eluted by adding 2 mL of M followed by 6 mL of C:M (1:1 v/v). The eluate was dried under N_2, then dissolved in 200 μL of C:M:H_2O (60:30:8 v/v/v). A 10-μL aliquot was applied to an HPTLC plate (catalog number 1.05641.0001, Supelco, Thermo Fisher Scientific) and developed in C:M:0.25% $CaCl_2$ (60:35:8 v/v/v). Gangliosides were detected chemically by resorcinol-HCl (5) as blue-violet bands. An individual gangliosides mixture (catalog number 1065, Matreya/Cayman Chemical Company) and a monosialoganglioside mixture (catalog number 1508, Matreya/Cayman Chemical Company) were applied as standards on the same HPTLC plate. The HPTLC plate was imaged on the Amersham Imager 680 (Cytiva, Global Life Sciences Solutions USA) for quantification using ImageQuant TL software.

The absolute amount of GM2 ganglioside in a brain sample was determined based on a standard curve generated with known quantities of GM2 ganglioside run on the same plate.

Western blotting. Cells or tissues were homogenized in RIPA Lysis and Extraction buffer (Thermo Fisher Scientific) supplemented with 1% SDS, HALT protease inhibitor cocktail (Thermo Fisher Scientific), and HALT phosphatase inhibitor cocktail (Thermo Fisher Scientific). Samples were incubated 30 min on ice and later spun at 14,000 x *g* at 4° C for 10 min. Equal amounts of lysed protein samples (30 μg) were resolved on a 4–12% Bis-Tris gel (Thermo Fisher Scientific), then transferred to nitrocellulose membranes using the iBlot2 Blotting System (Thermo Fisher Scientific). The membranes were blocked in 5% non-fat dry milk, 0.05% Tween 20 in Tris-buffered saline, then probed with the primary antibody. The primary antibodies used were HEXA mouse monoclonal antibody (D-2, catalog number sc-376777, Santa Cruz Biotechnology) to detect mouse α-subunit β-hexosaminidase A expression, Cas9 N-terminal mouse monoclonal antibody (clone 7A9, catalog number MAC133, Millipore Sigma), CD68 rabbit monoclonal antibody (clone E307V, catalog number 97778, Cell Signaling), GFAP mouse monoclonal antibody (clone GA5, catalog number 3670, Cell Signaling), Gpnmb rabbit monoclonal antibody (clone E7U1Z, catalog number 90205, Cell Signaling) and Hmox1 mouse monoclonal antibody (clone 2D10A5, catalog number 66743-1-Ig, Proteintech). Primary antibodies were detected by incubation with an HRP-conjugated goat anti-mouse IgG antibody (catalog number AP134P; MilliporeSigma) or HRP-conjugated goat anti-rabbit IgG antibody (catalog number AP132P; MilliporeSigma). Membranes were re-probed with anti-mouse β-actin antibody (monoclonal AC-15, HRP-conjugated, catalog number ab49900, Abcam) to provide a loading control. Membranes were developed using chemiluminescence reagents (ECL

Prime Western Blotting System, catalog number GERPN2232, from MilliporeSigma) and visualized using the Amersham Imager 680 imager.

Supplemental Table 1. Amplicon genomic position and primer sets for sequencing of the 18 off-target sites selected from CIRCLE-seq.

**Off-target loci amplified and sequenced**			
**Site ID**	**Position of Alignment with sgRNA**	**Forward Primer (5'-3')**	**Reverse Primer (5'-3')**
**On-Target[1]**	chr15:72350500-72350522	GGTATCCGTGTGCTTGCAGAG	TAAGGACCAAGGCTGGGATATGC
**Off Target 1[2]**	chr13:110128376-110128398	TCACAAGCCCCTTTGCTGAA	ACCTGGGGTTGAATTGAAGGT
**Off Target 2[2]**	chr10:26401711-26401734	CCCATCCCGTATGAGGTGTG	GGTTATGTTCATGGGTAGGGGT
**Off Target 3[2]**	chr13:28015626-28015649	CAGAGGATGCAAAGCCAGGA	GTAAAATGGATGGCCCCCGA
**Off Target 4[2]**	chr11:28079396-28079419	GAGGACCGCTCCCACATTTA	GGCTTTCAGACCTGATTCAGC
**Off Target 5[2]**	chr12:17938003-17938026	GGTCAGCACACTCACCTGAT	TGAGATCTGATAAGATCATTGAGGGG
**Off Target 6[2]**	chr3:62602869-62602892	ACTGGAACGTCTCCTCTCCA	GCCTGCTTCCTGGTACTTCC
**Off Target 7[2]**	chr10:35580376-35580399	GGCCACACCTGTCACATCAA	GCCAGGCAGCAATTCTGAAA
**Off Target 8[2]**	chr8:32160359-32160382	ACTGTGTGACTAGGAACTGAACT	CAAACCCCAGGCCCACAATA
**Off Target 9[2]**	chr4:140347829-140347852	GTGGGAAAGGGGCAATGGTA	AGAGGACTGTGTCTCCTAAGGA
**Off Target 10[2]**	chr8:129917244-129917267	GCGGTGAAAGTGGACATCCT	TCCTGATTACAACTCTCAGATACTT
**Off Target 11[2]**	chr10:124275861-124275884	CCCAGTTCTAGACATTGGGCA	GGTGGCCAAGGAGAGGTTTA

**Off-target loci that failed amplification**				
**Site ID**	**Primer set**	**Position of Alignment with sgRNA**	**Forward Primer (5'-3')**	**Reverse Primer (5'-3')**
**Off-target 12[2]**	**1**	chr3:37223973-37223996	CACACTCTGCCTCCACATCT	GGAGATCAAGGCTGCAGTGA
	**2**	chr3:37223973-37223996	CTGCCTCCACATCTTCTGCA	CTGCAGTGAGCCGAGATCAT
**Off-target 13[2]**	**1**	chr1:39046652-39046675	GGGTTTCACCGTGTTAGCCA	AGAAACAGCAGTAGCCATGGT
	**2**	chr1:39046652-39046675	TCACCGTGTTAGCCAGGATG	GATGCTGTGTGTGTGTGTGT
**Off-target 14[2]**	**1**	chr4:14992219-14992242	TTTAGTCAAGATGCATCACTTTTCT	AGTAGAGACCTCAGAGAAAAAGCA
	**2**	chr4:14992219-14992242	TGCATCACTTTTCTCTGCCT	AGAGACCTCAGAGAAAAAGCATCT
**Off-target 15[2]**	**1**	chr9:20097667-20097689	GGACTGGGTATCTGATTCTCCC	TGGGTGACAGAGCAAGACTC
**Off-target 16[2]**	**1**	chr11:37848963-37848985	TCGTGTGTGTGTAATCCATCAGT	CACACACACACACACACAGC
**Off-target 17[2]**	**1**	chr1:113660519-113660542	CTCTCTAAGCTGGGCCTCACT	TTTTTGTTTACAAAGAGCAGACCA
**Off-target 18[2]**	**1**	chr11:129624799-129624822	AGTACAGTGGTTGTCTCGGA	GGCAACAAACACTGCTAGTTGT

[1] On-target refers to the exact sequence recognized on *HEXA* by the LOTS-sgRNA.
[2] Sites identified as potential off-target loci by CIRCLE-seq (Supplemental Figure 1A)

Supplemental Table 2. Number of control-treated and ABE-treated LOTS mice used for evaluation of base-editor treatment.

Control-treated and ABE-treated LOTS mice			
End of life		21 weeks old	
ABE-treated	Control-treated	ABE-treated	Control-treated
6 females	3 females	6 females	7 females
4 males	4 males	18 males	18 males

Supplemental table 3. Sequence of DNA donor templates used for mouse model generation.

ssDNA oligo	Sequence
***HEXA* exon 7 c.805G**	GAAAGTTGTAGAGGAACACACACAATGGTGCTGCCGCATGCAGACATGCACACTCACCAAAAGAAAGCAGTAG<br>ATGTGATAACTTTGGGGAAATTTTATCATAGTTTTATCATAATAGACTCTTGAAGAAGTCACCAGTACACATGGGCT<br>TCGGACGAGTGTTCTAAAATGTGGGCATTTTGAGTATCTTCTACTCTGCTAGCTTTCAGGAAGTGTGAACCTGAA<br>GGGTGTCTTGTGCCTTTCAGGGGTCCTACAACCCTGTCACCCACATCTACACAGCACAGGATGTGAAGGAGGT<br>CATTGAATACGCACGGCTCCGGGGTATCCGTGTGCTTGCAGAGTTTGACACTCCTGGCCACACTTTGTCCTGG<br>GGACCAGGTAAGAATGATGTCTGGGACCAGAGGGACTCTGCTTGTTATGCTCAGAGTGAAGCTTCAGGGCACT<br>GGCTCATGGAAGTGGCATATCCCAGCCTTGGTCCTTAGAAGAATGTTTTCCATCGACTTCTTCCACCTGGGAATT<br>TAGATAGGAAGACATAGGTAAAGCAAGAAAGTGGCTCAAGTGAGCCAGCTCCTGCTTCGGAAGTTACTCCTAAG<br>GGTGTCTTGGCCCCAGAGTCTTCCTGCCTGGGCTGCTC
***HEXA* exon 7 c.805A**	GAAAGTTGTAGAGGAACACACACAATGGTGCTGCCGCATGCAGACATGCACACTCACCAAAAGAAAGCAGTAG<br>ATGTGATAACTTTGGGGAAATTTTATCATAGTTTTATCATAATAGACTCTTGAAGAAGTCACCAGTACACATGGGCT<br>TCGGACGAGTGTTCTAAAATGTGGGCATTTTGAGTATCTTCTACTCTGCTAGCTTTCAGGAAGTGTGAACCTGAA<br>GGGTGTCTTGTGCCTTTCAGGGGTCCTACAACCCTGTCACCCACATCTACACAGCACAGGATGTGAAGGAGGT<br>CATTGAATACGCACGGCTCCGGGGTATCCGTGTGCTTGCAGAGTTTGACACTCCTGGCCACACTTTGTCCTGG<br>GGACCAAGTAAGAATGATGTCTGGGACCAGAGGGACTCTGCTTGTTATGCTCAGAGTGAAGCTTCAGGGCACT<br>GGCTCATGGAAGTGGCATATCCCAGCCTTGGTCCTTAGAAGAATGTTTTCCATCGACTTCTTCCACCTGGGAATT<br>TAGATAGGAAGACATAGGTAAAGCAAGAAAGTGGCTCAAGTGAGCCAGCTCCTGCTTCGGAAGTTACTCCTAAG<br>GGTGTCTTGGCCCCAGAGTCTTCCTGCCTGGGCTGCTC

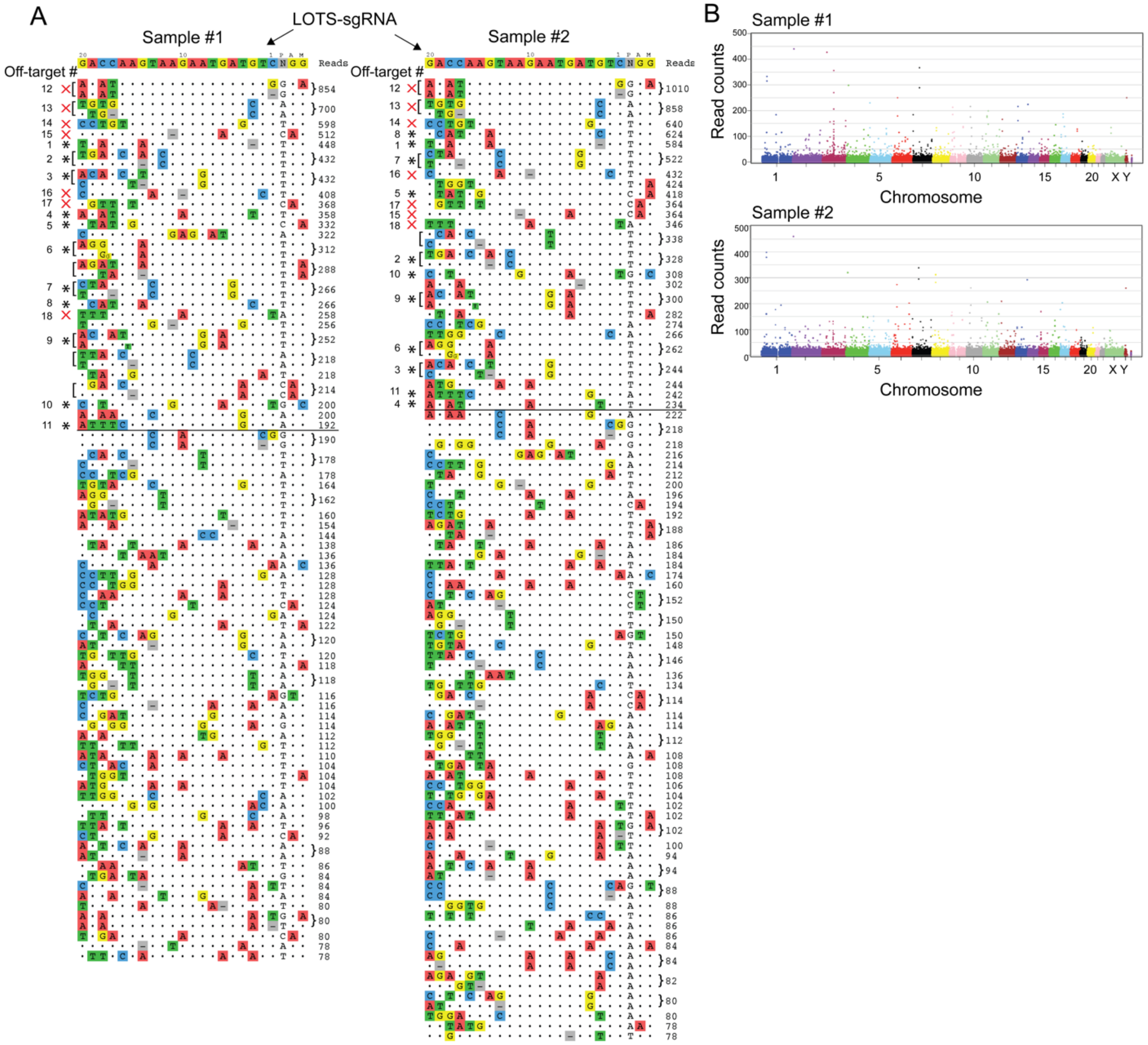

Supplemental Figure 1. CIRCLE-seq analysis of human DNA using *S. pyogenes* Cas9 nuclease and the LOTS-sgRNA to identify genome-wide off-target candidate sequences. (**A**) CIRCLE-seq read sequences and counts obtained from Cas9-treated unaffected-human genomic DNA (n=2, identified as samples #1 and #2). From the top 25 loci hits for each sample (demarcated by the horizontal line), 18 candidates were

found in common in both samples (their corresponding off-target numbers from Supplemental Table 1 are indicated). From those, 11 candidates (marked with *) were subjected to targeted amplicon sequencing (data shown in Figure 1E). The remaining 7 loci were not analyzed due to lack of PCR amplification (marked with a red X). LOTS-sgRNA protospacer and PAM sequences are shown at the top. Counts for each read are shown on the right. Changes from the LOTS-sgRNA protospacer and PAM sequences are noted by colored base, and conserved bases are marked with a dot. Deleted bases are indicated by a gray dash. Bracketed reads are sequences that the program aligned to the same position in the genome although are not totally identical. (**B**) CIRCLE-seq–detected off-target sequences from Cas9-treated genomic DNA from non-affected fibroblasts were visualized as Manhattan plots and were organized by chromosomal position and the read count associated with that position. Data showed that the detection of the off-target sites was evenly distributed throughout the genome, since no statistically significant differences were found. Statistical significance was analyzed using Student's t-test run in R.

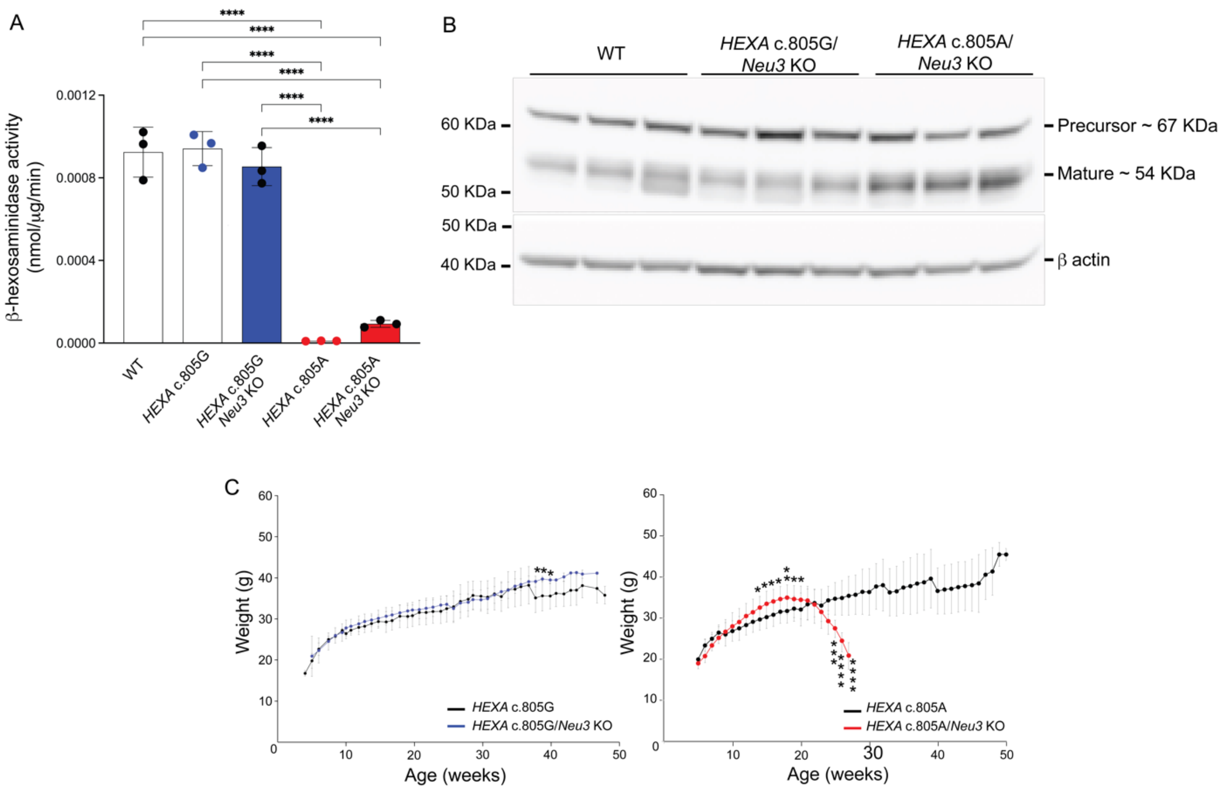

Supplemental Figure 2. LOTS mice have reduced β-hexosaminidase activity in the brain and body-weight decline. (**A**) β-Hexosaminidase α-subunit–specific activity in mouse brain. Data are expressed as mean ± SD (n=3 for each genotype; each dot represents data for 1 mouse). All mice were males, 23–28 weeks of age. WT, WT C57BL/6 mice. Statistical significance was determined by one-way ANOVA with Bonferroni correction, **** p<0.0001. (**B**) Western-blot analysis of β-hexosaminidase A α-subunit expression in mouse brain. The precursor and mature forms are indicated. All mice were males, 23–28 weeks of age, n=3 per genotype. WT, WT C57BL/6 mice. β-Actin was used as a loading control. (**C**) Body-weight progression for male *HEXA* c.805G and *HEXA* c.805G/*Neu3* KO mice (left) and *HEXA* c.805A and *HEXA*

c.805A/*Neu3* KO mice (right) determined weekly. Data are expressed as mean ± SD for each time point (n=11 for *HEXA* c.805G; n=11 for *HEXA* c.805G/*Neu3* KO; n=10 for *HEXA* c.805A; n=10 for *HEXA* c.805A/*Neu3* KO). Statistical significance was determined by Student's *t*-test. * $p<0.05$, ** $p<0.01$, *** $p<0.001$, **** $p<0.0001$.

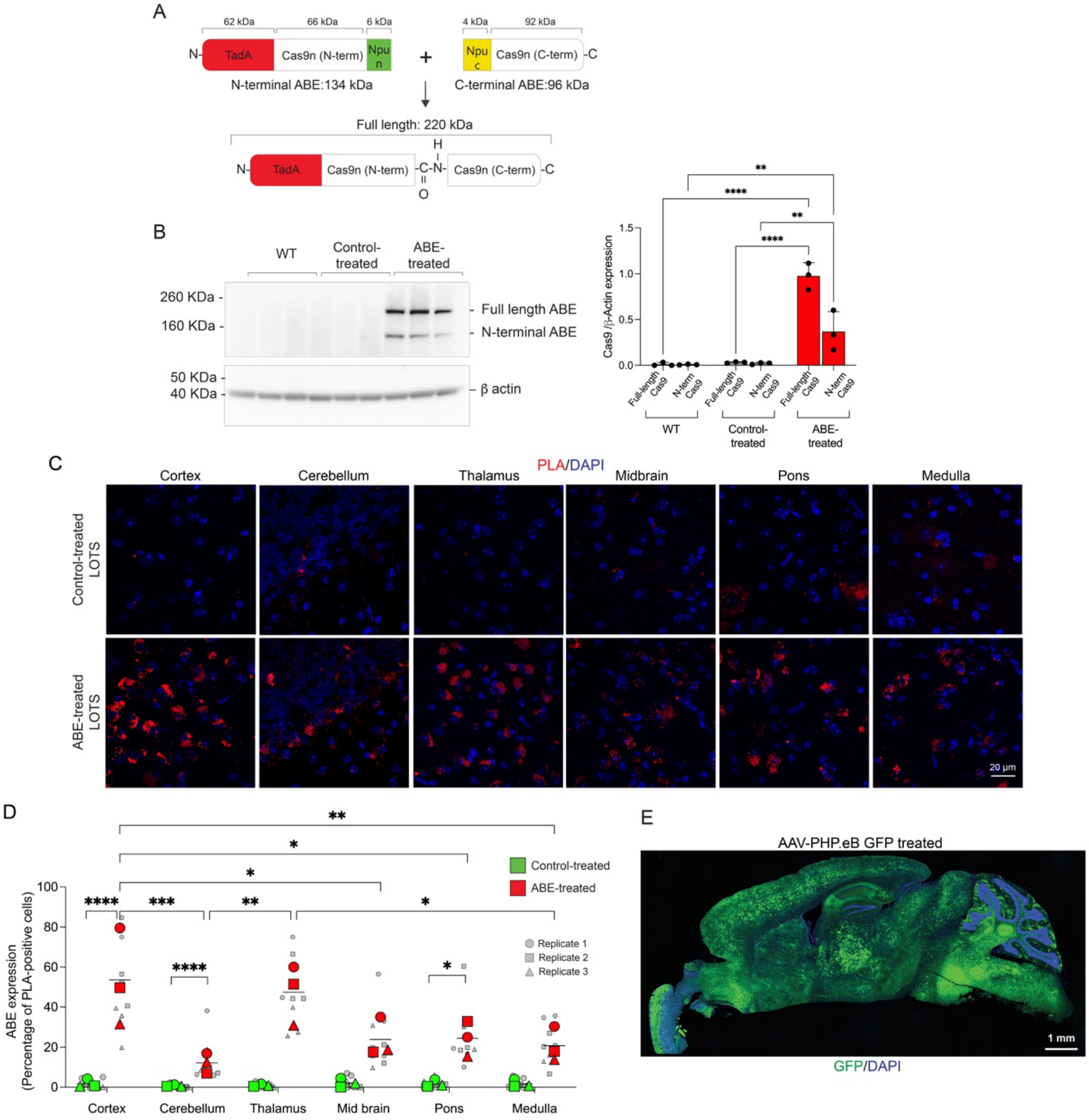

Supplemental Figure 3. ABE expression in ABE-treated LOTS brain.

WT mice, control-treated LOTS mice, and ABE-treated LOTS mice at 21 weeks of age were euthanized and their brains harvested and sectioned (n=4 for each genotype or treatment group, mixed males and females). The AAV-treated mice each received 2.4 ×

10^{12} vg. (**A**) Schematic of the formation of full-length base editor by protein splicing. Npu-n: N-terminal fragment of the Npu DnaE intein; Npu-c: C-terminal fragment of the Npu DnaE intein. (**B**) Analysis of the expression of full-length base editor protein in brain of ABE-treated brain lysates by Western blot using an N-terminal specific Cas9 antibody (top). β-actin was used as loading control. n=3 mice per group with quantification of the ABE protein (bottom). Data are shown band intensity normalized by β-actin. Statistical significance was determined by two-way ANOVA with Bonferroni correction. **p<0.01, **** p<0.0001. (**C** and **D**) Expression of full-length base editor in ABE-treated LOTS brains determined by PLA. (**C**) Representative 40x images of cerebral-cortex, thalamus, cerebellum, midbrain, pons and medulla from control-treated and ABE-treated brains. Sections were labelled with N-terminal and C-terminal Cas9-specific antibodies followed by PLA detection (red) and counterstained with DAPI (blue). n=3 mice per group. (**D**) Quantification of PLA^+ $DAPI^+$ cells as a percentage of total $DAPI^+$ nuclei. Small gray symbols represent image-level measurements (technical replicates); large colored symbols indicate per-mouse means (biological replicates). Cells were manually counted using FIJI software. Statistical significance was determined by two-way ANOVA with Bonferroni correction. *p<0.05, **p<0.01, *** p<0.001, **** p<0.0001. (**E**) Representative sagittal section image of a control-treated brain at 21 weeks old.

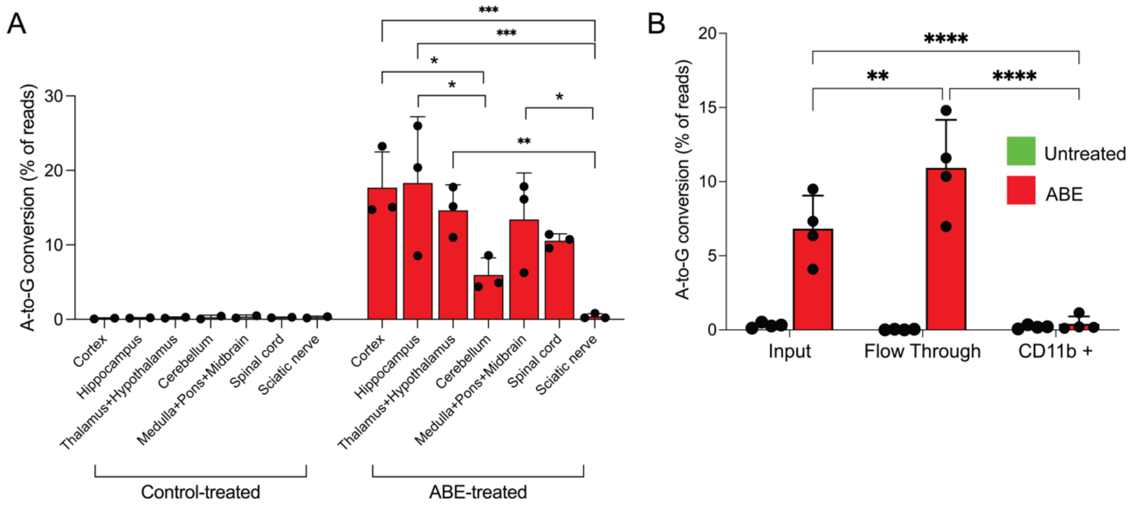

Supplemental Figure 4. Efficiency of LOTS mutation correction by ABE treatment. (**A**) Efficiency of A-to-G conversion in different brain regions of 21-weeks old ABE-treated (n=3) and control-treated (n=2) mice. The AAV-treated mice each received 2.4 × 10^{12} vg. Genomic DNA was purified from cortex, hippocampus, thalamus and hypothalamus, cerebellum, medulla+pons+midbrain combined, spinal cord, and sciatic nerve, and subjected to amplicon next-generation sequencing. *HEXA* c.805A-to-G conversion was expressed as mean percentage of A-to-G conversion ± SD from the total next-generation sequencing reads. Each dot represents data for 1 mouse. Statistical significance was determined by one-way ANOVA with Bonferroni correction. * p<0.05, ** p<0.01, *** p<0.001. (**B**) Correction of the *HEXA*.c.805A mutation by base editing in microglia. On-target base-editing efficiency was determined in genomic DNA purified from microglia isolated from brain of 26-week-old ABE-treated LOTS mice. Total brain genomic DNA (input), genomic DNA purified from cells in the flow through, and genomic DNA from CD11b-positive microglia cells ($CD11b^+$) were subjected to amplicon next-generation sequencing. *HEXA* 805c.A-to-G conversion was expressed as mean

percentage ± SD from the total next-generation sequencing reads obtained (n=4 mice per group). Each dot represents data for 1 mouse. Statistical significance was determined by two-way ANOVA with Bonferroni correction. **$p<0.01$, **** $p<0.0001$.

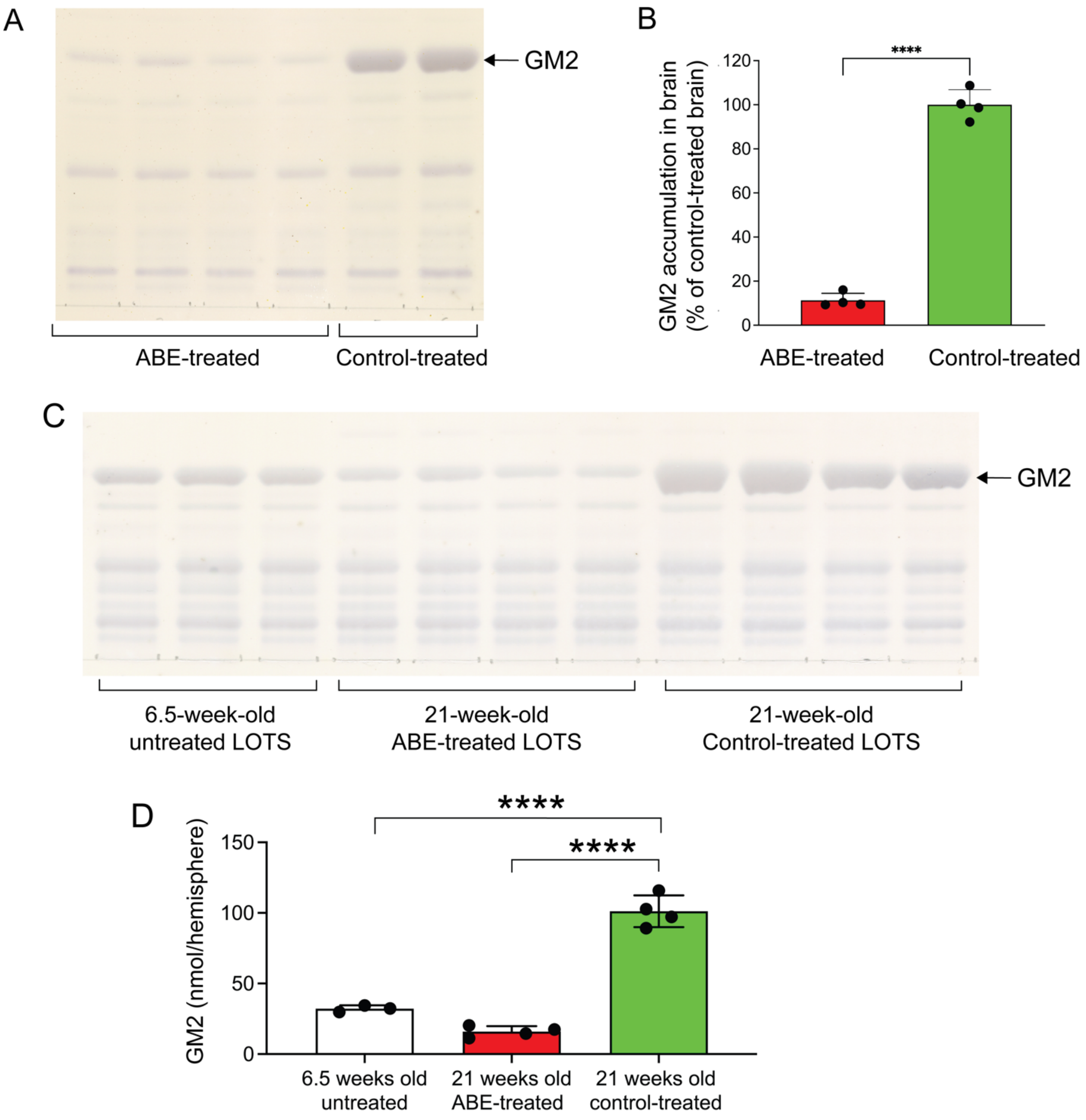

Supplemental Figure 5. Analysis of brain gangliosides in control-treated and ABE-treated LOTS mice. (**A and B**) Base-editor treatment reduces brain GM2 ganglioside accumulation in LOTS mice relative to control treated. Brain gangliosides were isolated from 21-week-old ABE-treated LOTS mice and control-treated LOTS mice and analyzed by HPTLC. The AAV-treated mice each received 2.4×10^{12} vg. (**A**)

Representative HPTLC analysis of brain gangliosides. The position of the GM2 ganglioside standard is indicated by the arrow. (**B**) Quantification of GM2 ganglioside band intensities from HPTLC analysis. Each lane corresponds to 0.5% of the total gangliosides extracted from 1 mouse brain hemisphere. Data are expressed as GM2 ganglioside level in ABE-treated LOTS mouse brains as mean percentage of the GM2 ganglioside level in control-treated LOTS mouse brains (set at 100%) ± SD (n=4 mice for each treatment group, all female mice). Each dot represents data for 1 mouse. Statistical significance was determined by Student's *t*-test. **** $p<0.0001$. (**C and D**) Base-editor treatment reduces brain GM2 ganglioside accumulation in LOTS mice relative to pre-treatment levels. Brain gangliosides were isolated from 6.5-week-old untreated LOTS mice, 21-week-old ABE-treated LOTS mice compared to 21-week-old control-treated LOTS mice and analyzed by HPTLC. (**C**) Representative HPTLC analysis of brain gangliosides. Each lane corresponds to 0.5% of the total gangliosides extracted from 1 mouse brain hemisphere. The position of the GM2 ganglioside standard is indicated by the arrow. (**D**) Quantification of GM2 ganglioside band intensities from HPTLC analysis. Data are expressed as mean nmols of GM2 ganglioside per brain hemisphere ± SD (n=3 for 6.5-week-old untreated LOTS mice; n= 4 for 21-week-old ABE-treated LOTS mice). Each dot represents data for 1 mouse. Statistical significance was determined by one-way ANOVA with Bonferroni correction, **** $p<0.0001$.

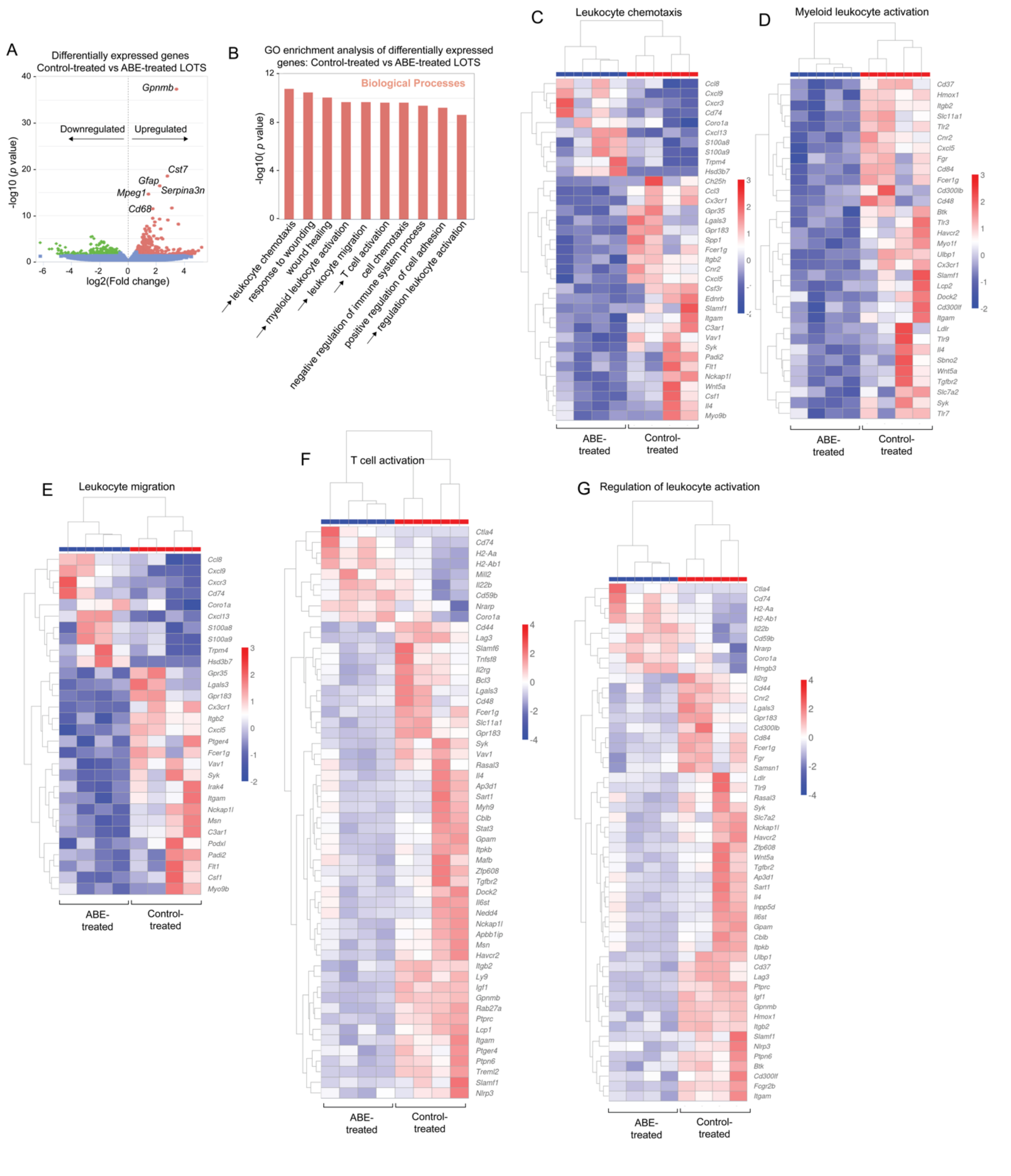

Supplemental Figure 6. Base-editor treatment reduces expression of neuroinflammation markers in brain of LOTS mice. Control-treated and ABE-treated

LOTS mice at 21 weeks of age were euthanized, brains harvested, and RNA isolated for RNA-seq analysis. The AAV-treated mice each received 2.4×10^{12} vg. Data were analyzed using the cloud platform NovoMagic (Novogene) (n=4 for each group). (**A**) Volcano plot showing significantly differentially expressed genes in brains from control-treated LOTS mice compared with ABE-treated LOTS mice. Genes significantly upregulated ($\log_2$ fold change > +1) in control-treated LOTS mice are shown in red. Genes significantly downregulated ($\log_2$ fold change > −1) are shown in green. Genes with expression levels not significantly different or between $\log_2$ fold change −1 and +1 are shown in blue. (**B**) Top 10 GO biological process genes that are differentially expressed in control-treated LOTS mice compared with ABE-treated mice. Arrows indicate the gene sets shown in Supplemental Figure 6, C–G. (**C–G**) Heatmaps of RNA-seq expression Z-scores computed for genes in individual control-treated and ABE-treated LOTS mice from the 5 biological process categories indicated with arrows in Supplemental Figure 6B. Genes that are differentially expressed ($p < 0.05$, $|\log_2(\text{foldchange})| > 1.5$) between control-treated and ABE-treated LOTS mice are shown. Each column on the heatmaps corresponds to an individual mouse. Note that the 4 ABE-treated brain samples clustered separately from the ones corresponding to control-treated mice.

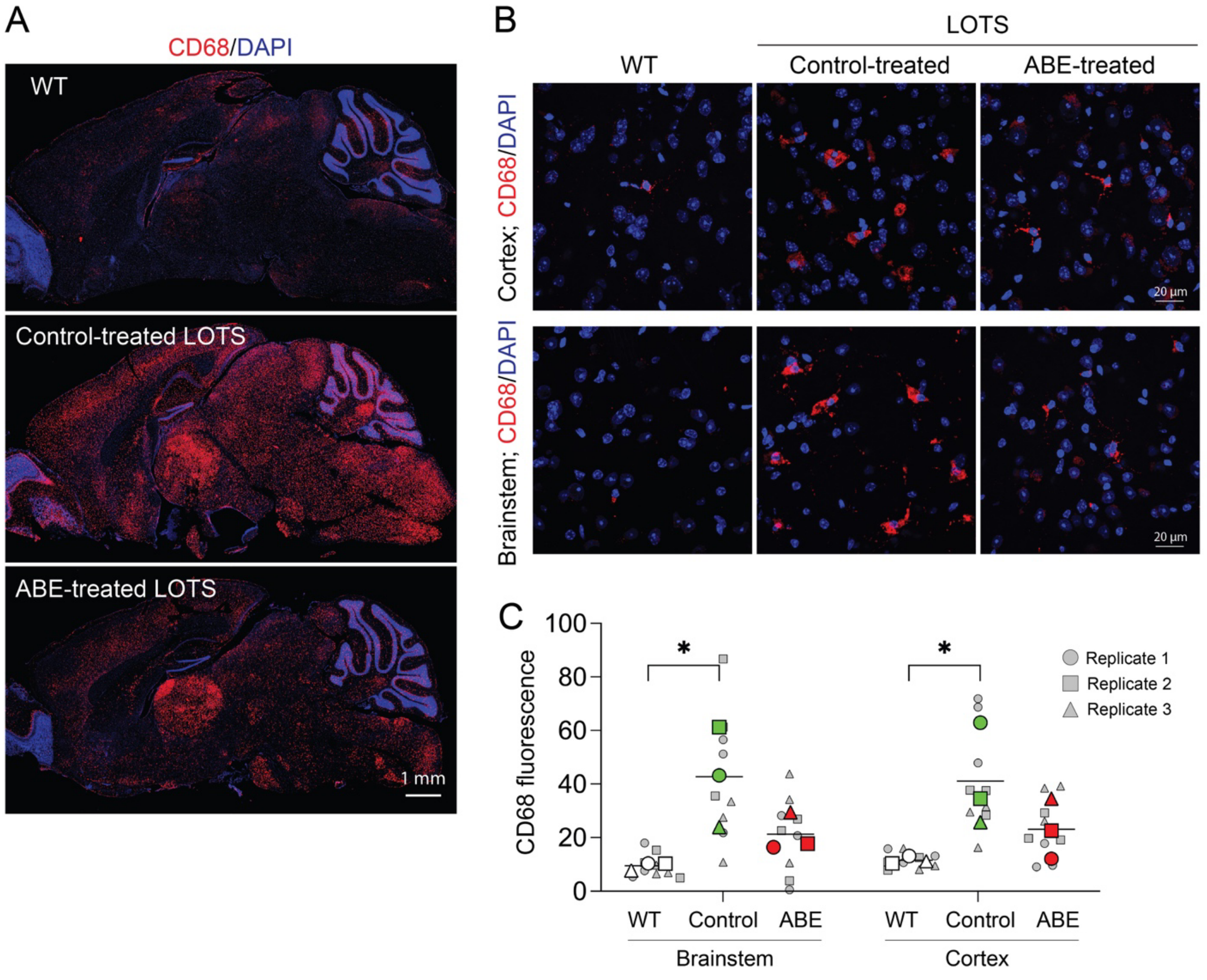

Supplemental Figure 7. Base-editor treatment reduces expression of CD68 in brain of LOTS mice. WT, control-treated LOTS, and ABE-treated LOTS mice were euthanized at 21 weeks of age, and sagittal brain sections were immunostained. The AAV treated mice each received 2.4 × 10^{12} vg. (**A**) Representative images brain sections stained with anti-CD68 antibody (red) and counterstained with DAPI (blue). (**B**) Representative 40x images of cerebral cortex (top panels) and brainstem (bottom panels) stained with anti-CD68 antibody (red) and counterstained with DAPI (blue). (**C**) Quantification of CD68 fluorescence intensity in brainstem and cortex area. Small gray

symbols represent image-level measurements (technical replicates); large colored symbols indicate per-mouse means (biological replicates). Statistical analysis was performed using a mixed-effects model with Tukey's correction.* $p<0.05$. n=3 for WT; n=3 for control-treated group; n=4 for ABE-treated.

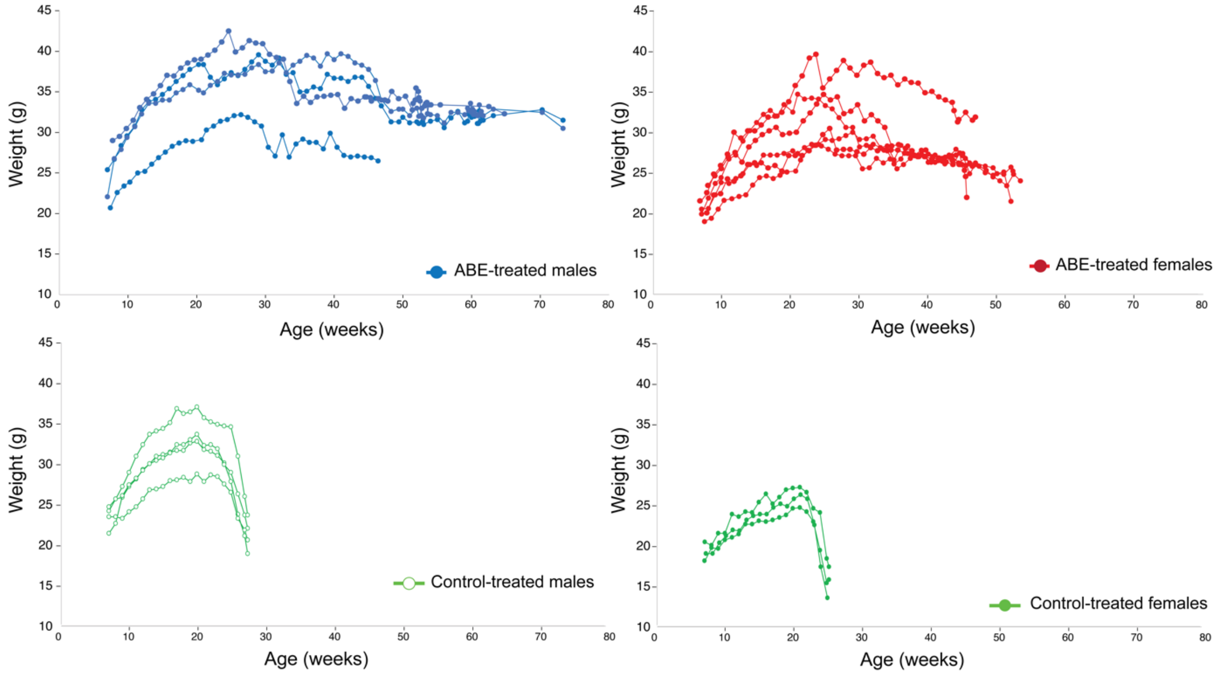

Supplemental Figure 8. Base-editor treatment mitigates weight loss in LOTS mice. Body-weight progression for individual male and female ABE-treated LOTS mice (top panels) and control-treated LOTS mice (bottom panels) determined weekly. These data were averaged and presented in Figure 6A (n=4 for ABE-treated males; n=6 for ABE-treated females; n=4 for control-treated males; n=3 for control-treated females).

References

1. Tsai SQ, Nguyen NT, Malagon-Lopez J, Topkar VV, Aryee MJ, and Joung JK. CIRCLE-seq: a highly sensitive in vitro screen for genome-wide CRISPR-Cas9 nuclease off-targets. *Nat Methods.* 2017;14(6):607-614.
2. Lazzarotto CR, Nguyen NT, Tang X, Malagon-Lopez J, Guo JA, Aryee MJ, et al. Defining CRISPR-Cas9 genome-wide nuclease activities with CIRCLE-seq. *Nat Protoc.* 2018;13(11):2615-2642.
3. Duffy HBD, Byrnes C, Zhu H, Tuymetova G, Lee YT, Platt FM, et al. Deletion of Gba in neurons, but not microglia, causes neurodegeneration in a Gaucher mouse model. *JCI Insight.* 2024;9(21):e179126.
4. Folch J, Lees M, and Sloane Stanley GH. A simple method for the isolation and purification of total lipides from animal tissues. *J Biol Chem.* 1957;226(1):497-509.
5. Svennerholm L. Chromatographic Separation of Human Brain Gangliosides. *J Neurochem.* 1963;10:613-623.
