# Supplementary material for: CNS-targeted base editing of the major late-onset Tay-Sachs mutation alleviates disease in mice
Source: J Clin Invest. 2025 Jun 17;135(16):e183434. doi: 10.1172/JCI183434 (PMC12352896; doi:10.1172/JCI183434)

7/25/24-

Uncropped Fig1E-HexA

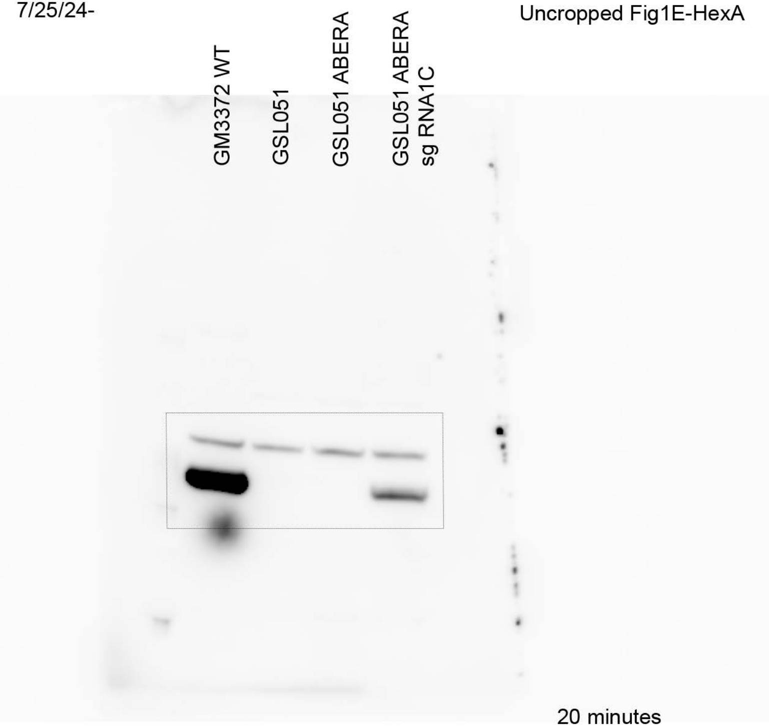

20 minutes

Hex A (D-2) (Santa Cruz Biotechnology)- 1:1000 primary antibody in 5% milk overnight at 4C
secondary antibody- 1:2500 anti-mouse in 5% milk for 1 hr at room temperature

30ug protein/each lane

7/26/24-

Uncropped Fig1E-Actin

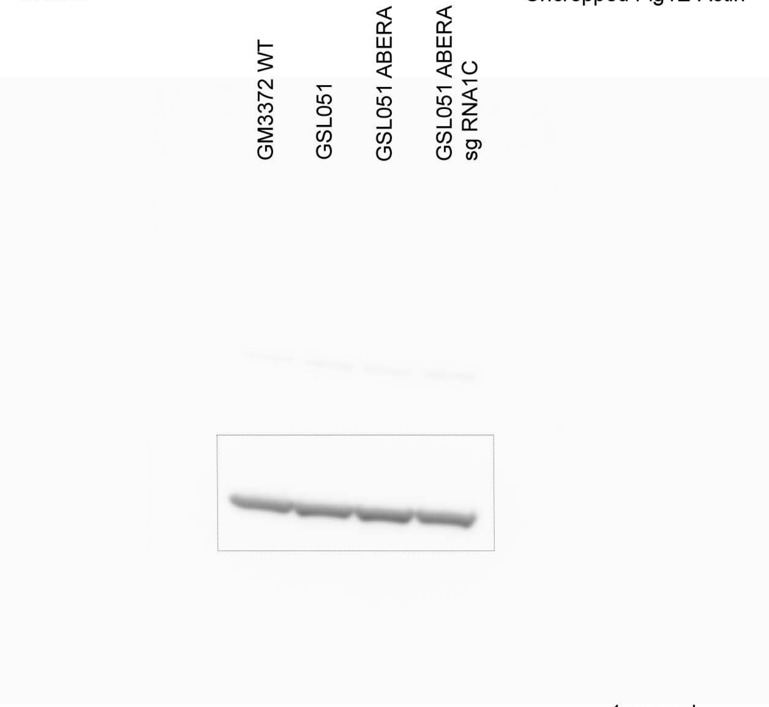

1 second

B-actin- 1:20000 primary antibody in 5% milk for 45 minutes at room temperature

30ug protein/each lane

Uncropped Figure 2D

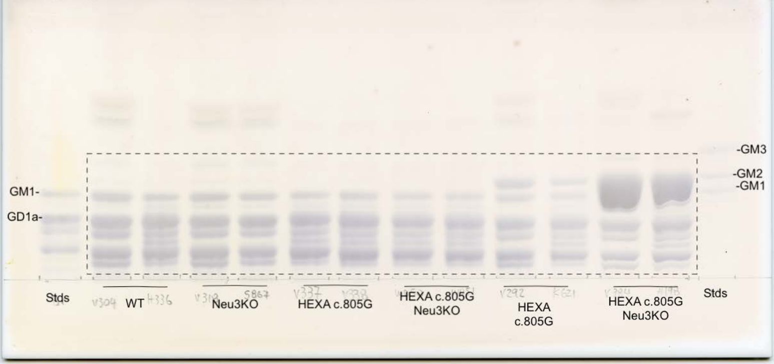

7/31/24-

Uncropped Fig 5H-CD68

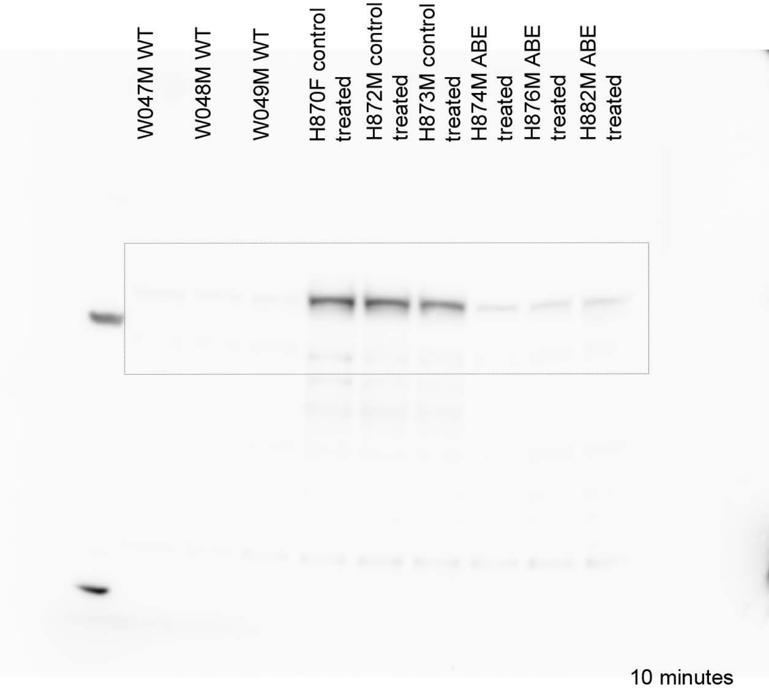

CD68 (Cell Signaling Technology #97778)- 1:1000 primary antibody in 5% BSA (0.1% Tween 20) overnight at 4C
secondary antibody- 1:3000 anti-rabbit in 5% milk (0.1% Tween 20) for 1 hr at room temperature

30ug protein/each lane

8/8/24- Uncropped Fig 5i-GFAP

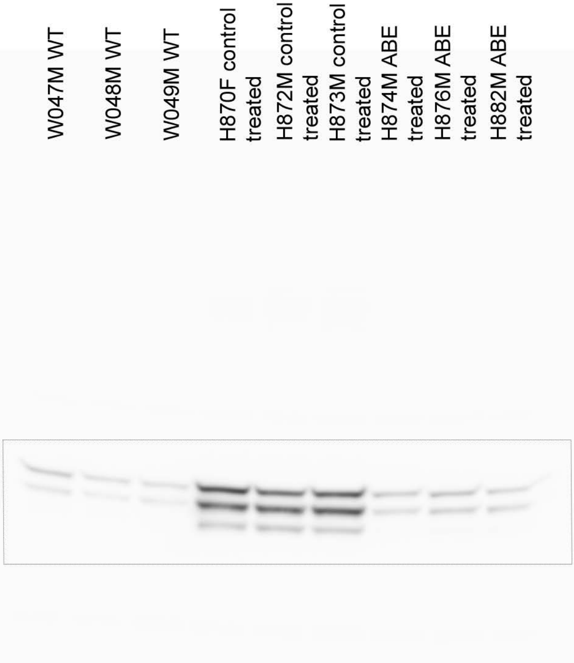

1 second

GFAP (Cell Signaling Technology #3670)- 1:50000 primary antibody in 5% milk (0.1% Tween 20) overnight at 4C
secondary antibody- 1:2500 anti-mouse in 5% milk (0.1% Tween 20) for 1 hr at room temperature

30ug protein/each lane

8/1/24-

Uncropped Fig 5H-Actin

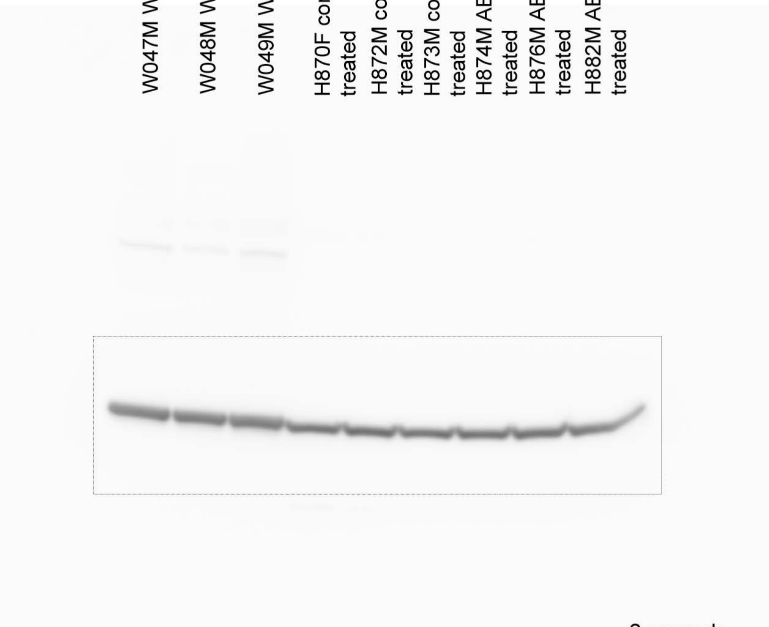

2 seconds

B-actin (Sigma Aldrich)- 1:20000 primary antibody in 5% milk for 45 miuntes at room temperature

30ug protein/each lane

8/9/24-

Uncropped Fig 5i-actin

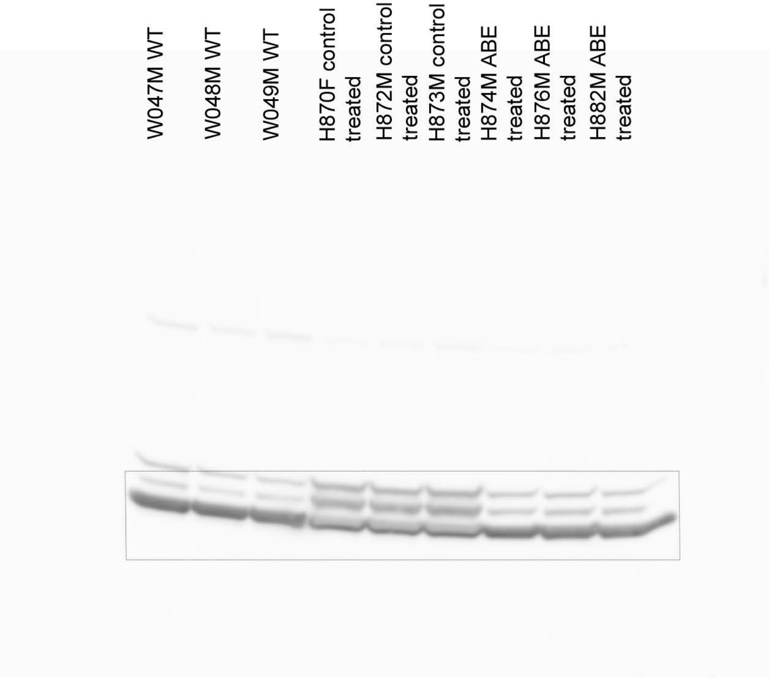

4 seconds

B-actin (Sigma Aldrich)- 1:20000 primary antibody in 5% milk for 45 minutes at room temperature

30ug protein/each lane

8/6/24-

Uncropped Fig 5J-Gpnmb

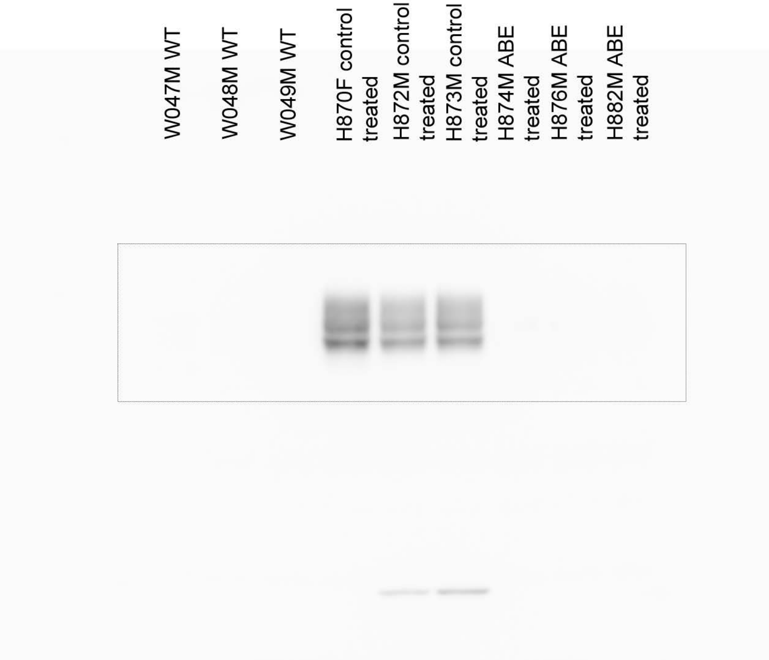

29 seconds

Gpnmb (Cell Signaling Technology #90205)- 1:1000 primary antibody in 5% BSA (0.1% Tween 20) overnight at 4C
secondary antibody- 1:3000 anti-rabbit in 5% milk (0.1% Tween 20) for 1 hr at room temperature

30ug protein/each lane

8/7/24-

Uncropped Fig 5J-Actin

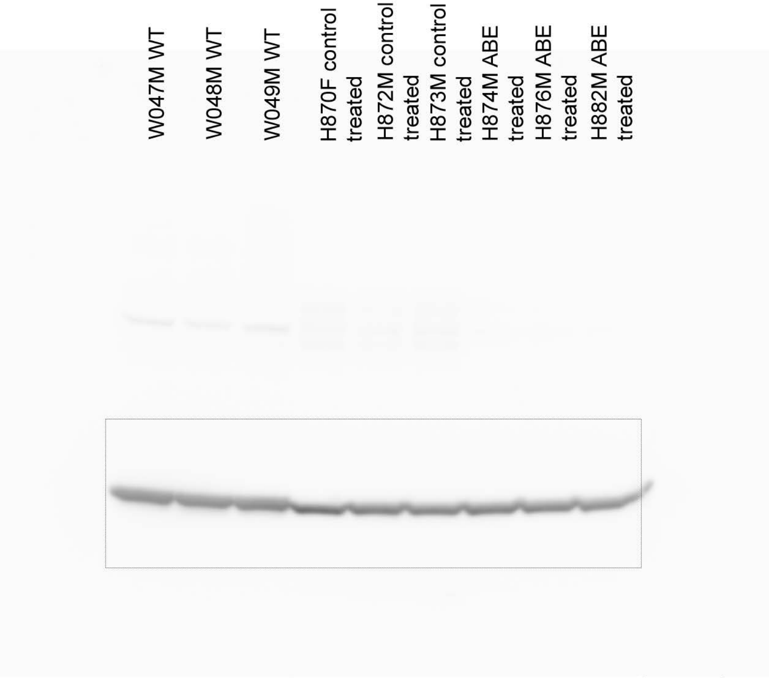

3 seconds

B-actin (Sigma Aldrich)- 1:20000 primary antibody in 5% milk for 45 minutes at room temperature

30ug protein/each lane

uncropped SuppFig 2B-HexA

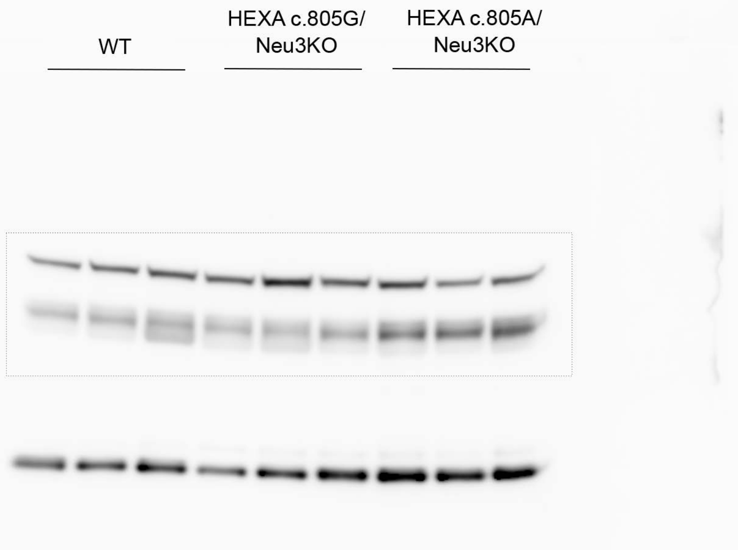

3 minutes

Hex A (D-2) (Santa Cruz Biotechnology)- 1:500 primary antibody in 5% milk overnight at 4C

secondary antibody- 1:2500 anti-mouse in 5% milk for 1 hr at room temperature

40ug protein/each lane

Uncropped Figs 7F and Suppl. Fig 5A

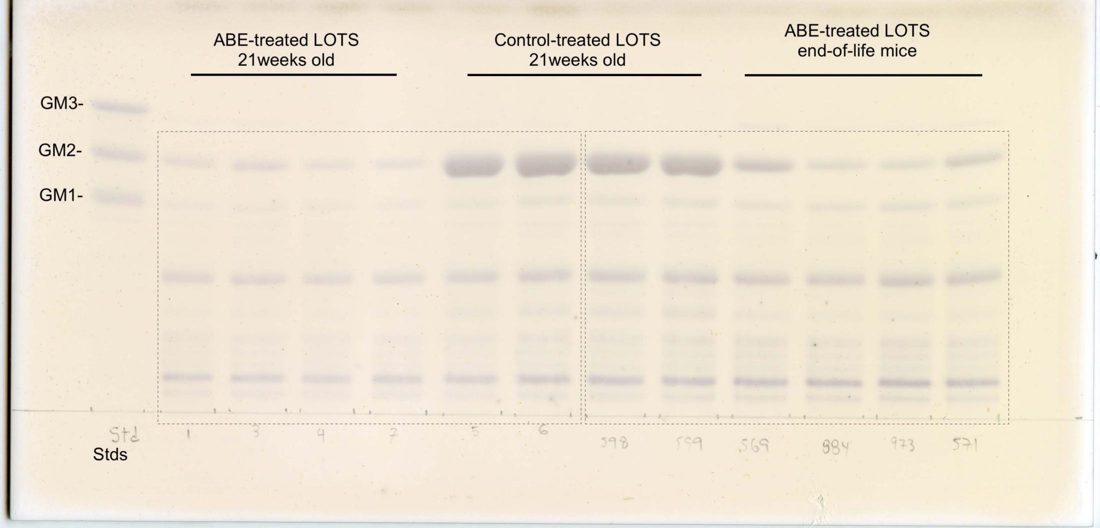

uncropped SuppFig 2B-actin

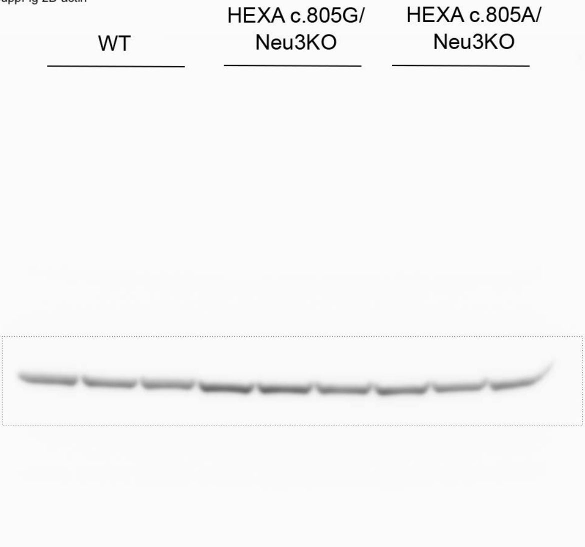

15 seconds

B-actin (Cell Signaling Technology)- 1:20000 primary antibody in 5% BSA (0.1% Tween20) for 45 minutes at room temperature

40ug protein/each lane

8/21/24-

Uncropped Suppl Fig 3B-Cas9

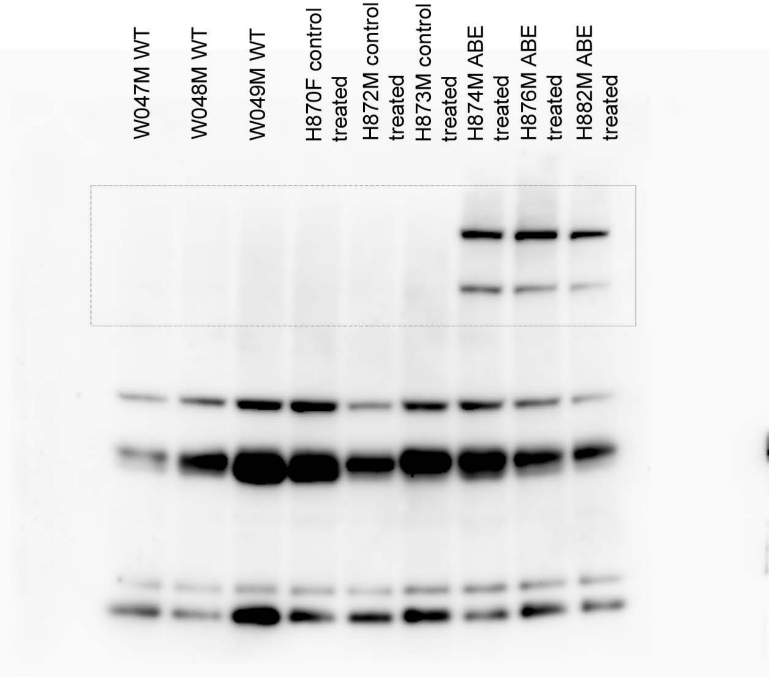

40 minutes

Cas9 (MilliporeSigma)- 0.5ug/ml primary antibody in 5% milk overnight at 4C
secondary antibody- 1:2500 anti-mouse in 5% milk for 1 hr at room temperature

30ug protein/each lane

8/22/24- Uncropped Suppl Fig 3B-actin

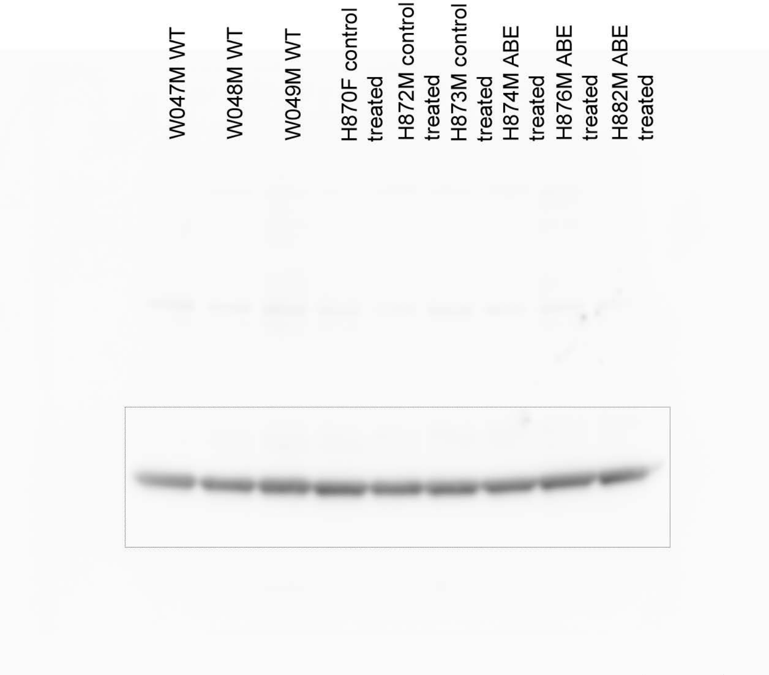

1 second

B-actin (Sigma Aldrich)- 1:20000 primary antibody in 5% milk for 45 minutes at room temperature

30ug protein/each lane

uncropped SuppFig 5C

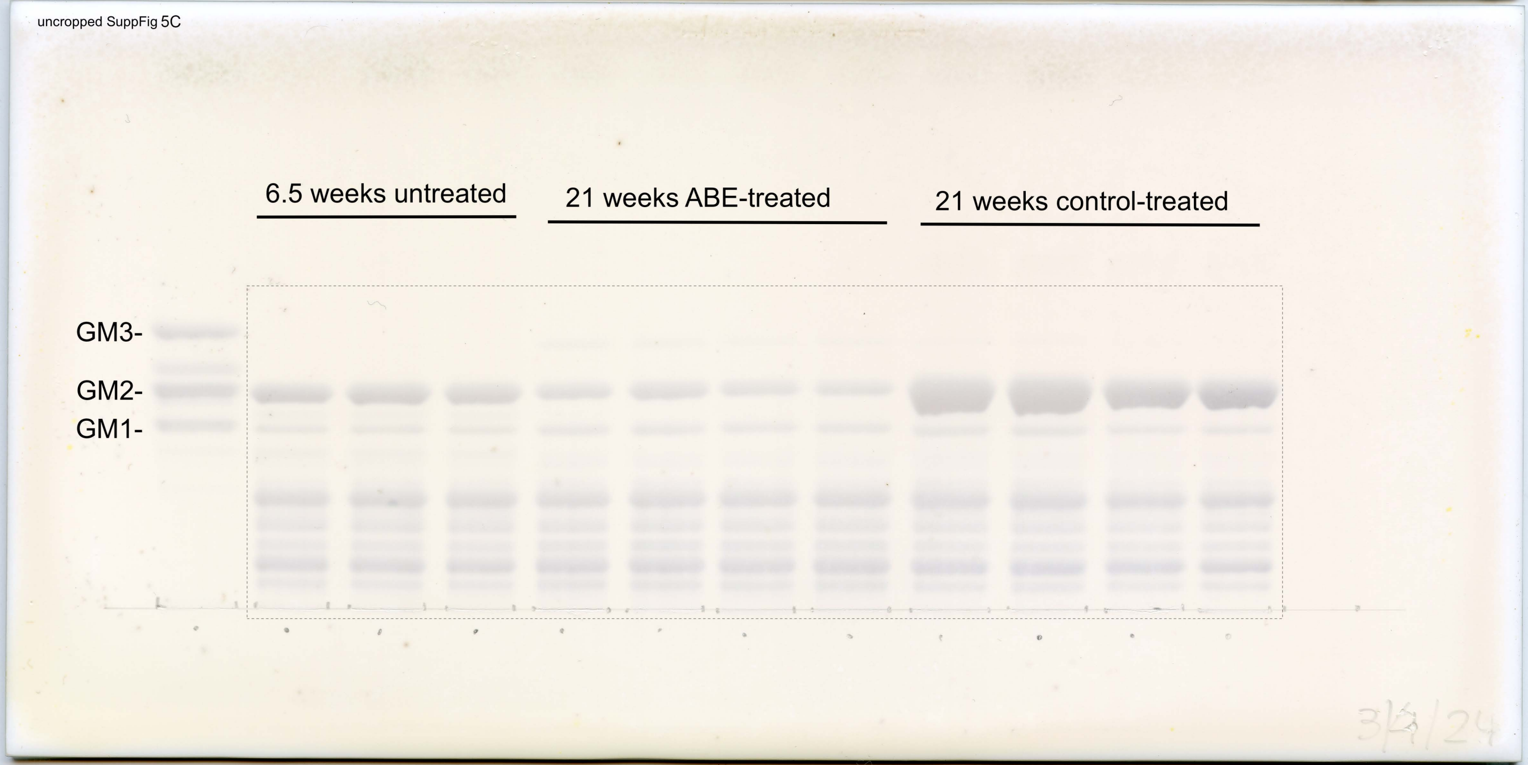

Supplement: Unedited blot and gel images [file jci-135-183434-s104.pdf]
